# Improvement of the Irrigation Scheme in the ORCHIDEE Land Surface Model and Impacts of Irrigation on Regional Water Budgets Over China

**DOI:** 10.1029/2019MS001770

**Published:** 2020-04-24

**Authors:** Z. Yin, X. H. Wang, C. Ottlé, F. Zhou, M. Guimberteau, J. Polcher, S. S. Peng, S. L. Piao, L. Li, Y. Bo, X. L. Chen, X. D. Zhou, H. Kim, P. Ciais

**Affiliations:** ^1^ Laboratoire des Sciences du Climat et de l'Environnement IPSL, CNRS‐CEA‐UVSQ Gif‐sur‐Yvette France; ^2^ Laboratoire de Météorologie Dynamique, IPSL UPMC/CNRS Paris France; ^3^ Sino‐French Institute for Earth System Science, College of Urban and Environmental Sciences Peking University Beijing China; ^4^ UMR 7619 METIS, IPSL, Sorbonne Universités, UPMC, CNRS, EPHE Paris France; ^5^ Key Laboratory of Tibetan Environment Changes and Land Surface Processes, Institute of Tibetan Plateau Research Chinese Academy of Sciences Beijing China; ^6^ CAS Center for Excellence in Tibetan Plateau Earth Sciences Beijing China; ^7^ State Key Laboratory of Hydrology‐Water Resources and Hydraulic Engineering, Center for Global Change and Water Cycle Hohai University Nanjing China; ^8^ Institute of Industrial Science The University of Tokyo Tokyo Japan

**Keywords:** irrigation model, paddy irrigation, irrigation water withdrawal, crops, local climate, total water storage

## Abstract

In China, irrigation is widespread in 40.7% cropland to sustain crop yields. By its action on water cycle, irrigation affects water resources and local climate. In this study, a new irrigation module, including flood and paddy irrigation technologies, was developed in the ORCHIDEE‐CROP land surface model which describes crop phenology and growth in order to estimate irrigation demands over China from 1982 to 2014. Three simulations were performed including NI (no irrigation), IR (with irrigation limited by local water resources), and FI (with irrigation demand fulfilled). Observations and census data were used to validate the simulations. Results showed that the estimated irrigation water withdrawal ( W) based on IR and FI scenarios bracket statistical W with fair spatial agreements ( r=0.68±0.07; p<0.01). Improving irrigation efficiency was found to be the dominant factor leading to the observed W decrease. By comparing simulated total water storage (TWS) with GRACE observations, we found that simulated TWS with irrigation well explained the TWS variation over China. However, our simulation overestimated the seasonality of TWS in the Yangtze River Basin due to ignoring regulation of artificial reservoirs. The observed TWS decrease in the Yellow River Basin caused by groundwater depletion was not totally captured in our simulation, but it can be inferred by combining simulated TWS with census data. Moreover, we demonstrated that land use change tended to drive W locally but had little effect on total W over China due to water resources limitation.

## Key Points

- A new irrigation module is developed in ORCHIDEE including drip, flood, and paddy irrigation
- Simulations can explain broad scale patterns and the trends of irrigation amount from census data
- Irrigation efficiency and land use change are key factors affecting the trend of irrigation

## 1 Introduction

Irrigation, accounting for 70% of global total water usage (FAO, 2019), contributed to more than 40% of food production increase in the past six decades (George et al., 2011). With the growth of population and economy, irrigated cropland area grew fourfold in the last century (Siebert et al., 2015), implying a huge increase of water demand (Hanasaki et al., 2008; Wada et al., 2016). Simultaneously, climate change, such as extreme events (Ben‐Ari et al., 2018; Seneviratne et al., 2014) and CO ${}_{2}$ fertilization (Osborne, 2016), brings divergent impacts on soil moisture in agricultural systems, which indirectly affects water management. To meet the growing demand for food production and enhance the resilience of agricultural system against climate change under water scarcity, we need to understand the role that irrigation played in agriculture and its interactions with the terrestrial water cycle and climate.

The primary aim of irrigation is to compensate a water deficit that crops need for growth. The amount of irrigation applied to crops is not only determined by the demand from different cultivar types and climate conditions but also by irrigation techniques and water availability (Leng et al., 2017; Savva et al., 2002; Wada et al., 2016). Nazemi and Wheater (2015a, 2015b) summarized different approaches to assess irrigation demand in land surface models (LSMs) and global hydrological models (GHMs) and suggested that the computation of irrigation demand should be associated with specific techniques, as well as local farming habits, to provide more precise estimations. For instance, paddy irrigation is applied for lowland paddy rice in traditional rice cultivation Asian countries (Bouman et al., 2007; Salmon et al., 2015; Wada et al., 2016) not only to alleviate water stress but also to suppress weeds and pests, accounting for 34–43% of the global irrigation water withdrawal ( W) (Bouman et al., 2007). In some studies, W is estimated to match the full demand from crops (Guimberteau et al., 2012; Haddeland et al., 2014; Hanasaki et al., 2018; Nazemi & Wheater, 2015a; Wada et al., 2014). However, constrained by available water resources, actual irrigation amount could be smaller. Surface water (e.g., rivers and lakes) and shallow groundwater are commonly considered in GHMs and LSMs as sources for irrigation (Leng, Tang, et al., 2015; Leng, Huang, et al., 2015; Zeng et al., 2017). However, due to the lack of corresponding local and regional data about groundwater use, nonrenewable groundwater is difficult to be simulated by these models (Scanlon et al., 2018). Consequently, W could be underestimated in arid and semiarid regions, where groundwater use is known to be significant (Leng, Huang, et al., 2015; Pokhrel et al., 2015; Rodell et al., 2018; Zeng et al., 2017).

The impacts of irrigation on climate are non‐negligible (Guimberteau et al., 2012; Haddeland et al., 2014; Lobell, 2007; Lobell et al., 2008; Sacks et al., 2009; Thiery et al., 2017). At local scale, irrigation should promote evapotranspiration (ET), which in turn decreases surface temperature ( $T_{s}$) that acts as a negative impact on ET (Seneviratne et al., 2010). Chen and Jeong (2018) demonstrated that irrigation induced nighttime warming linked to the larger heat capacity of wet soils is more significant than the daytime evaporative cooling in the North China Plain, suggesting the complexity of the ET‐temperature interactions under irrigation. At continental scale, irrigation can alleviate heat extreme events (Thiery et al., 2017), while it may reinforce deadly heatwaves by increasing atmospheric humidity during hot days in future climate scenarios (Kang & Eltahir, 2018). Moreover, continuous irrigation may postpone summer monsoon and reduce precipitation from May to July (Guimberteau et al., 2012). Therefore, besides water and energy balances, physical, physiological, and social effects should also be considered in irrigation assessments.

Recent studies used GHMs or LSMs to assess irrigation amount and its impact on water resources and local climate. For example, by improving the groundwater pumping module in CLM4 (Community Land Model), Leng, Huang, et al. (2015) estimated surface water and groundwater withdrawals under climate change. Zeng et al. (2017) investigated the responses of hydrological cycle and local climate to groundwater depletion showing that irrigation can either increase or decrease precipitation by enhancing evapotranspiration or by suppressing summer monsoon, respectively. These studies did improve our understanding of the complex effects of irrigation on water cycle, but they ignored associated biophysical and anthropogenic processes that can substantially influence irrigation variability. For example, as part of climate change, effect of CO ${}_{2}$ rise is missing, which may decrease irrigation demand by increasing water use efficiency (de Boer et al., 2011). The impact of land use change on irrigation and water cycle was revealed significant (Foley et al., 2005; Kueppers et al., 2007; Vicente‐Serrano et al., 2019), but it is rarely considered in the recent model studies as well. Moreover, irrigation demand is strongly affected by crop species and irrigation techniques, but few models are able to simulate such processes explicitly. Leng et al. (2017) provided a comprehensive assessment of global irrigation patterns by considering specific crop types and irrigation methods. However, rice, as an important staple crop, was not taken into account as well as associated paddy irrigation.

With the economy growth and technique improvement, China experienced fast irrigated area expansion by 51% since the 1980s (Zheng et al., 2004). It is reported that until 2017, $6.78\times 10^{8}$ m ${}^{3}$ cropland area are irrigated by $3.77\times 10^{11}$ m ${}^{3}\cdot$year ${}^{-1}$ accounting for 62.3% of total water usage (http://www.stats.gov.cn/tjsj/ndsj/2018/indexch.htm). In this study, we develop a new irrigation module in a physical‐based land surface model ORCHIDEE‐CROP (ORganizing Carbon and Hydrology in Dynamic EcosystEms, Wang, 2016) with a crop module for wheat, maize, and rice to simulate the patterns of irrigation demand in China. ORCHIDEE‐CROP accounts for most known biophysical responses of vegetation to climate change as well as crop phenological stages (Wang, 2016). The irrigation model considers the most used irrigation techniques in China, especially paddy irrigation for rice. Both surface water and renewable groundwater are simulated as sources of water supply. Due to the low quality of the irrigation data in China (http://www.fao.org/nr/water/aquastat/irrigationmap/index40.stm), we do not use the fraction of groundwater withdrawal from Siebert et al. (2010) to trace the source of irrigation water as Leng, Huang, et al. (2015) but only assess total irrigation amount. Moreover, to provide reasonable estimation in regions where croplands are widely irrigated by nonrenewable groundwater, we introduce an unlimited irrigation mode that allows irrigation demand fulfilled without considering water balance. More importantly, a new land use data set subdividing croplands into wheat, maize, rice, and other crops has been established based on remote sensing and census data from 1982 to 2016 in order to provide precise assessment of irrigation under land use change. Three simulations are designed with different scenarios in this study: NI (no irrigation), IR (irrigation limited by local water resources), and FI (irrigation demand fulfilled). Through these simulations we aim to (1) estimate irrigation demand and water withdrawal in China and validate them by census data and (2) investigate how irrigation affects water and energy budgets.

In section 2, the new irrigation model will be explicitly described, following introduction to data sets used as input or for validation. After the experiment design shown in section 2.3, we validate simulated irrigation withdrawal by census data and demonstrate how irrigation affects water and energy fluxes over China in section 3. Limitations of model, comparisons with parallel works, and perspectives are finally discussed in section 4.

## 2 Model, Data, and Simulation Protocol

### 2.1 New Irrigation Module in ORCHIDEE

Irrigation processes were introduced in the standard version of ORCHIDEE by de Rosnay et al. (2003) and further developed by Guimberteau et al. (2012). However, these early developments were made for hydrological modeling without considering the nature of cropland managements. For example, water and energy budgets of croplands are shared with other plant functional types (e.g., forests and grasslands) in the same grid, which makes the simulated irrigation to be distributed uniformly among different vegetation types. We have now developed a new irrigation scheme in the crop branch of ORCHIDEE model called ORCHIDEE‐CROP (Wang, 2016; Wang et al., 2017; Wu et al., 2016). Crops are modeled with specific equations for phenology and carbon allocation, previously calibrated for wheat, maize, and rice at site and regional scale. Global gridded applications of crops with ORCHIDEE have proven its capability for regional/global applications (e.g., Müller et al., 2017, 2018; Porwollik et al., 2017; Schauberger et al., 2017; Schewe et al., 2019).

**Figure 1:**
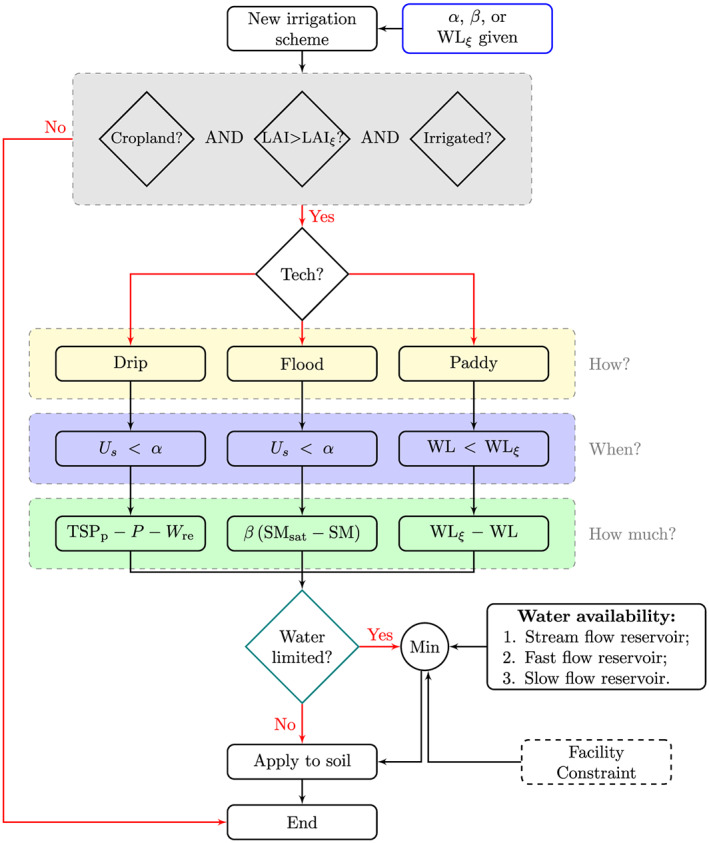
Flowchart of the new irrigation scheme in ORCHIDEE. Blue‐border rectangle contains pre‐defined parameters. Three conditions highlighted by gray rectangle determine whether irrigation will be applied. Irrigation time (blue rectangle) and water demand (green rectangle) associate with irrigation technique (yellow rectangle). The teal‐border diamond determines whether water balances are taken into account. Dashed‐line rectangle indicates that the functions are under developing and not applied in this study. The explanation of mathematical variables can be found in section 2.1.

The new irrigation scheme aims to represent the main irrigation types encountered all over the world and especially in China. The first principle was to supply water to irrigated crops to alleviate water stress for crop growth and its consequences for water and energy budget. The flowchart of this new module is given in Figure 1. The three main components (colored rectangles in Figure 1) are the simulation of the timing or irrigation events, the supply from renewable water resources, and the description of three different techniques, namely, drip, flood (no pond), and paddy (with pond) irrigation. The choice of the irrigation technique is first pre‐defined by the user (yellow rectangle in Figure 1). Then the model calculates the condition to trigger irrigation (blue rectangle) and the water amount needed by the crop (the green rectangle). In the case of drip and flood techniques, irrigation starts when the simulated hydric stress on plant photosynthesis and transpiration ( $U_{s}$) gets below a threshold α. $U_{s}$ is calculated as the sum of relative soil moisture (SM) weighted by root density in each soil layer and can vary between $U_{s}=0$ representing the wilting point and $U_{s}=1$ representing an absence of stress (de Rosnay et al., 2002). In case of paddy irrigation, irrigation starts when the paddy water level (WL) gets below a threshold value WL ${}_{\xi }$, which represents the target minimum WL desirable for this type of practice.

The irrigation requirement ( $I_{\text{req}}$), depending on irrigation techniques, is calculated at the ORCHIDEE model time step (e.g., each 30 min). In the case of drip irrigation, $I_{\text{req}}$ is calculated from the difference between potential transpiration and the sum of precipitation and reinfiltration, as in Guimberteau et al. (2012). In the case of flood irrigation, $I_{\text{req}}$ is estimated by a fraction ( β) of the difference between saturated soil moisture SM ${}_{\text{sat}}$ and actual soil moisture SM. In the case of paddy irrigation, $I_{\text{req}}$ is calculated as the amount of water needed to maintain WL above the target value WL ${}_{\xi }$. This is summarized by

(1) $$I_{\text{req}}=\left\{\begin{array}{ll} {\text{TR}}_{\text{p,crop}}-P-W_{\text{re}} & \text{if}\;U_{s}<\alpha \text{, for drip irrigation},\\ \beta \left({\text{SM}}_{\text{sat}}-\text{SM}\right) & \text{if}\;U_{s}<\alpha \text{, for flood irrigation},\\ {\text{WL}}_{\xi }-\text{WL} & \text{if WL}<{\text{WL}}_{\xi }\text{, for paddy irrigation}, \end{array}\right.$$

where $I_{\text{req}}$ [mm] is the irrigation requirement or the water demand; TR ${}_{\text{p,crop}}$ [mm] is the potential transpiration of specific crop; P[mm] is precipitation; $W_{\text{re}}$ [mm] is reinfiltration; SM ${}_{\text{sat}}$[mm] is the saturated soil moisture; SM [mm] is the current soil moisture; WL ${}_{\xi }$ [mm] is the target paddy water level; WL [mm] is the current water level; and α [ −] is the pre‐defined $U_{s}$ threshold.

The $I_{\text{req}}$ can only be supplied by renewable fresh water resources in each grid cell that include the stream, fast, and slow pools as defined in the routing scheme of ORCHIDEE (Guimberteau et al., 2012), with the stream reservoir ( $V_{1}$) being streamflow; the fast reservoir ( $V_{2}$) surface runoff; and the slow reservoir ( $V_{3}$) deep drainage. $I_{\text{req}}$ is tentatively met by using in priority $V_{1}$ then $V_{2}$ and finally $V_{3}$ as water supply sources. In this scheme, the water mass balance is conserved, but the demand cannot exceed the supply at each time step. If demand exceeds supply, irrigation stays at the maximum possible supply value. Simultaneously, we stipulate that the applied irrigation amount ( $I_{\text{app}}$) at each time step cannot exceed a threshold $I_{\max}$ defined by

(2) $$I_{\text{app}}=\min\left(I_{\text{req}},\overset{3}{\underset{i=1}{\sum }}V_{i},I_{\max}\right),$$

where $V_{i}$ is water reservoirs in ORCHIDEE and $I_{\max}$ is set to 0.5 mm $\cdot \Delta t^{-1}$ (equivalent to 24 mm ·day ${}^{-1}$) to avoid undesired runoff generated when the irrigation exceeds the soil infiltration capacity.

The irrigation module can also calculate irrigation in the case where the demand is entirely fulfilled, to provide an idealized potential condition where crops are not often constrained by renewable freshwater resources. In this case, the model will create water for irrigation in excess of the supply and break water conservation. In practice, such missing water could be supplied by groundwater. This upper limit of the irrigation amount is used in this study to estimate the potential yield of crop and to define the maximum irrigation amount needed in irrigated regions.

After estimating $I_{\text{app}}$, water supplied by renewable freshwater pools is applied. Unlike precipitation, irrigated water is not intercepted by crop canopies. Drip irrigation directly inserts water into the soil root zone, while flood and paddy irrigation sets water on the surface of the cropland. Different from paddy irrigation, flood irrigation is not forced to maintain water in a pond. Thus, runoff may occur when irrigation rate is larger than the infiltration rate. In the case of paddy irrigation, when WL >0, soil evaporation is equal to potential evaporation. A maximum height of the paddy water level above ground is defined by WL ${}_{\max}$. Precipitation falling upon the paddy accumulates in the flooded paddy to keep the WL between WL ${}_{\xi }$ and WL ${}_{\max}$, and the rest is lost by runoff. If the precipitation is not sufficient to keep the paddy water level above WL ${}_{\xi }$, additional water is given to the paddy from irrigation. When paddy rice has been harvested, the paddy model is turned off and the water balance is calculated by the standard model water balance equations. Note that WL does not influence crop photosynthesis and transpiration in the model.

One of the key functions of ORCHIDEE‐CROP that make our results significantly different from Guimberteau et al. (2012) is the inclusion of the crop module. In the standard version of ORCHIDEE, crop was treated as a productive grassland with a long growing season that makes possible irrigation to occur all year around. In the crop model, crops have specific planting dates, and their growing period is generally shorter for major cereal crops. Second, in Guimberteau et al. (2012), $I_{\text{req}}$ was constrained by an irrigation fraction of each grid cell, defined as the ratio of irrigated area over total cropland area. This assumption was not optimal because the water balance of rainfed and irrigated plants was inconsistently calculated for a single soil tile. This setting was unrealistic for crop modeling as it implies that rainfed crops also received irrigation water. In the new module, rainfed and irrigated crops grow on separated soil tiles, with independent calculation of water and energy budgets.

### 2.2 Data Sets

#### 2.2.1 China 15‐PFT Map

**Figure 2:**
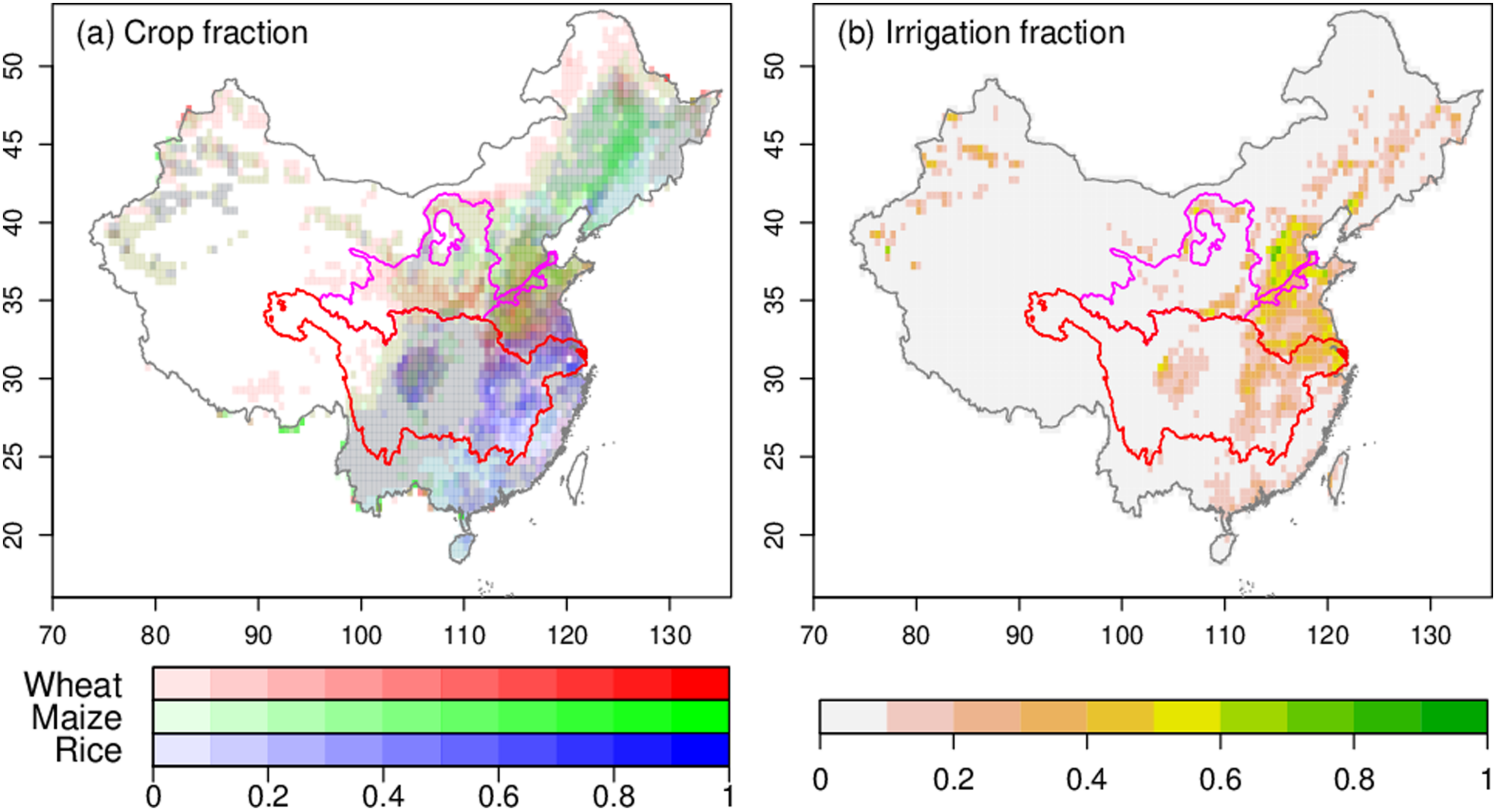
(a) Multi‐year averaged crop fraction based on the 15‐PFT map. Red, green, and blue indicate fraction of wheat, maize, and rice, respectively. (b) Irrigation fraction derived from GMIA.

The 15‐PFT (Plant Functional Type) map accounts for bare soil (PFT 1), woody plants (PFT 2–9), C3/C4 grasses (PFT 10–11), wheat (PFT 12), maize (PFT 13), rice (PFT 14), and other crops (PFT 15). Cropland area over China is categorized into four PFTs (wheat, maize, rice, and other crops). As main staple food and feed resources, wheat, maize, and rice account for 57.2% of China's cropland area. The spatial distribution of these crops was derived from the 1:1 million vegetation map of China (Figure 2a; separated crop fraction plots can be found in Figure S1 of the supporting information) for the year from 1982 to 2016, and temporal changes are obtained by increasing or decreasing the fraction of each grid cell with crops in each province to match provincial scale annual census data from National Bureau of Statistics.

#### 2.2.2 Irrigation Withdrawal, Efficiency, and Fraction

Annual irrigation withdrawal ( $W_{\text{stat}}$) data are available from 1990 to 2013 from water usage annual statistics at provincial scale. These data are used as an independent check of the amount of irrigation water calculated by ORCHIDEE. $W_{\text{stat}}$ should be larger than the actual irrigation amount because it is the water taken from the source and not that is actually delivered to cropland. Water loss during transport is non‐negligible and amounts to up to 61.6% of $W_{\text{stat}}$ in some regions. Thus, in the following, we add a water transport loss term to the simulated amount of irrigation given to crops, $I_{\text{sim}}$, in order to compare it to $W_{\text{stat}}$. The modeled amount corrected for transport loss, $W_{\text{sim}}$, is given by

(3) $$W_{\text{sim}}=I_{\text{sim}}\frac{f}{\epsilon },$$

where $W_{\text{sim}}$ [m ${}^{3}\cdot$year ${}^{-1}$] is simulated irrigation withdrawal retrieved by $I_{\text{sim}}$; $I_{\text{sim}}$ [m ${}^{3}\cdot$year ${}^{-1}$] the simulated irrigation amount; f [ −] the irrigation fraction; and ϵ [ −] an irrigation efficiency factor (Wisser et al., 2008). To estimate f, we used the Global Map of Irrigation Area (GMIA) version 5 from Siebert et al. (2005, 2013), which combines agricultural census data, land cover maps, and satellite images around the year of 2005 into three products: the area equipped for irrigation, the area actually irrigated, and the source of irrigation water. The quality of irrigated area data is reported to be poor in China (GMIA assessment report, http://www.fao.org/nr/water/aquastat/irrigationmap/index40.stm). The value of f was set to the area equipped for irrigation, and it is displayed in Figure 2b. The value of ϵ was set from statistical provincial ϵ in 2010. Besides, statistical national ϵ is available from 1978 to 2015.

#### 2.2.3 Observation‐Based Data Sets for the Evaluation of Water Budgets

##### 2.2.3.1 Gridded Evapotranspiration

There are numerous global gridded evapotranspiration (ET) data products derived from different algorithms (Mueller et al., 2013). Most of those ET products are observation‐based models that estimate potential ET (ET ${}_{p}$) by the Penman‐Monteith, Priestley‐Taylor, or other formulas. Then actual ET is scaled from ET ${}_{p}$ using other factors such as soil moisture. Thus if the soil moisture variable used in an ET model does not contain information on irrigation, this ET product is not suitable for evaluating our model simulations. We selected GLEAM v3.2a (Global Land Evaporation Amsterdam Model, Martens et al., 2017) and FLUXCOM (Jung et al., 2009) from the ET products listed by Mueller et al. (2013), because they account for irrigation, to some extent. The GLEAM model calculates ET with a two‐layer soil model assimilating top‐soil moisture from the European Space Agency Climate Change Initiative (ESA CCI) SM product. GLEAM should therefore account indirectly for irrigation. FLUXCOM ET upscales FLUXNET (global network of eddy covariance towers) observations by using LPJmL (Lund‐Potsdam‐Jena managed Land) simulations through a Model Tree Ensemble (MTE) method (Jung et al., 2009). Thus, FLUXCOM ET is a hybrid product using flux towers, climate and satellite data, and the LPJmL simulation results.

We selected two ET products that may contain the effect of irrigation: the Peking University (PKU) catchment water balance model from Zeng et al. (2014) which used a Multiple Tree Ensemble model and eddy‐flux towers for downscaling spatial and temporal patterns of ET within each river catchment and the SEBS ET data set from Chen, Su, et al. (2019) which retrieved ET by using the Surface Energy Balance System (SEBS) model (Su, 2002). The SEBS model predicts ET by simulating the canopy‐atmosphere turbulent physical processes (Chen, Massman, et al., 2019). Chen et al. (2013) enhanced the turbulent parameterization of SEBS specially for the bare soil and major land covers over the Tibetan Plateau. Using the optimized SEBS version, Chen, Su, et al. (2019) produced a moderate resolution (5 km) estimate of daily ET at the global scale, driven by instantaneous MODIS land surface temperature and ERA‐interim meteorological data. Self‐preservation of ET during the day (Gentine et al., 2011, 2007) was used as a foundation to calculate the daily‐integrated ET from instantaneous sensible and latent heat fluxes estimates. MODIS NDVI, LAI, canopy height, canopy fraction, land cover, albedo, and land surface temperature products were used for the global daily ET calculations. Detailed information on the four ET data products can be found in Table S1.

##### 2.2.3.2 Total Water Storage

GRACE (Gravity Recovery and Climate Experiment) total water storage (TWS) products prepared by three different groups (Luthcke et al., 2013; Save et al., 2016; Watkins et al., 2015) were used for evaluation. TWS in ORCHIDEE accounts for water mass from soil, snow, interception, paddy water, and the three water reservoirs of the river routing scheme mentioned in section 2.1. It does not account for groundwater extraction (which is a strong signal over some crop covered regions in China, Rodell et al., 2018), for artificial reservoirs, or for glaciers mass. Therefore, in our comparisons with GRACE fields, we removed the multi‐year mean TWS in both fields and compare only yearly anomalies and trends. But because groundwater depletion is not simulated in the model, we expect it to underestimate negative TWS trends in regions where groundwater is used for irrigation (Northern China).

In the Yangtze River Basin (YZRB), the contribution of dams to TWS variation is non‐negligible. Generally, dams start to store water at the end of the flooding season (mid or end of September) and release water next spring for downstream irrigation. The regulation capacity of artificial reservoirs on the Yangtze River is large enough to influence TWS seasonality at basin scale. In particular, the regulation capacity of the Three Gorges Dam (TGD) is 221.5 $\times 10^{8}$ m ${}^{3}$, which can influence TWS seasonality by as much as 12.3 mm in the YZRB (1.808 $\times 10^{6}$ km ${}^{2}$). However, information related to dam management is difficult to collect. We only had monthly water storage data of the TGD in 2016. Here we assumed that there was no significant difference of dam regulation across years, and we used the TGD water storage data to correct the seasonality of simulated TWS from 2003 to 2014.

### 2.3 Simulation Protocol

The new 15‐PFT (Plant Functional Type) map (see section 2.2.1) regridded at the resolution of 0.5° was used to prescribe land cover in ORCHIDEE from 1982 to 2014. The spatial distribution of crops planting date was based on phenological observations obtained from the Chinese Meteorological Administration (Wang et al., 2017). Site observations of planting dates were interpolated with a kriging algorithm based on distance between stations, which is on average about equal to 50 km. Each specific crop in each grid cell is prescribed with a uniform planting date. Crop rotation and multicropping were not included in this study.

Simulations were driven by GSWP3 (Global Soil Wetness Project Phase 3) atmospheric forcing (Kim, 2017), which appeared to be the best forcing to reproduce the spatial and temporal soil moisture variations in the last three decades over China (Yin et al., 2018). GSWP3 has 3‐hourly temporal and 0.5° spatial resolution with precipitation based on the bias corrected GPCC v6 (Global Precipitation Climatology Centre, Becker et al., 2013). The soil texture map used in this work is from Zobler (1986).

In ORCHIDEE, each grid cell is fractioned into seven soil tiles for 15 PFT grouped as in Table S2. Since our aim is to investigate the impact of irrigation on water budgets and crops evapotranspiration, a 20‐year spin‐up was performed first by repeating the GSWP3 forcing in 1982 in order to reach equilibrium of water pools. Then simulations were performed from 1982 to 2014 over a domain covering China ranging [70° E–136° E] × [16° N–54° N].

Three simulation experiments were performed: (1) simulation without irrigation (NI), (2) simulation with irrigation by available water (IR), and (3) simulation with irrigation demand fulfilled (FI). Due to the lack of accurate information on the irrigated area for each crop type, we assumed that all crop tiles (containing different fractions from PFT 12–14) were irrigated in IR and FI. Moreover, drip irrigation is not applied in these simulations because this technique is much less used in China and related census data are missing. Therefore, flood irrigation was set as the irrigation technique for wheat and maize, and both α and β in equation (1) were set to one. In addition to calculate $I_{\text{app}}$ with flood irrigation, deep drainage in the model (water losses from the deepest soil layer in 2‐m depth) was shut down for crop soil columns during the growing period. Paddy irrigation was applied to all rice soil tiles. The value of WL ${}_{\max}$ was set to 100 mm, and WL ${}_{\xi }=$ to 50 mm (Wada et al., 2014). A part of precipitation may be maintained in the paddy. The settings of the three experiments are summarized in Table 1. Moreover, to investigate impacts of land use change on W, another two simulations were launched by using the same settings as IR and FI, respectively, but with static PFT map of 1982.

**Table 1:** Setting of the Three Simulations

		Technique		Threshold	
Experiments' abbreviations	Irrigation	Wheat and maize	Rice	Wheat and maize	Rice
NI	No irrigation	—	—	—	—
IR	Irrigated by available water	Flood	Paddy	α=1; β=1	WL ${}_{\xi }=$50 mm
FI	Irrigation demand fulfilled	Flood	Paddy	α=1; β=1	WL ${}_{\xi }=$50 mm

## 3 Results

### 3.1 Spatial Patterns of Simulated Irrigation

**Figure 3:**
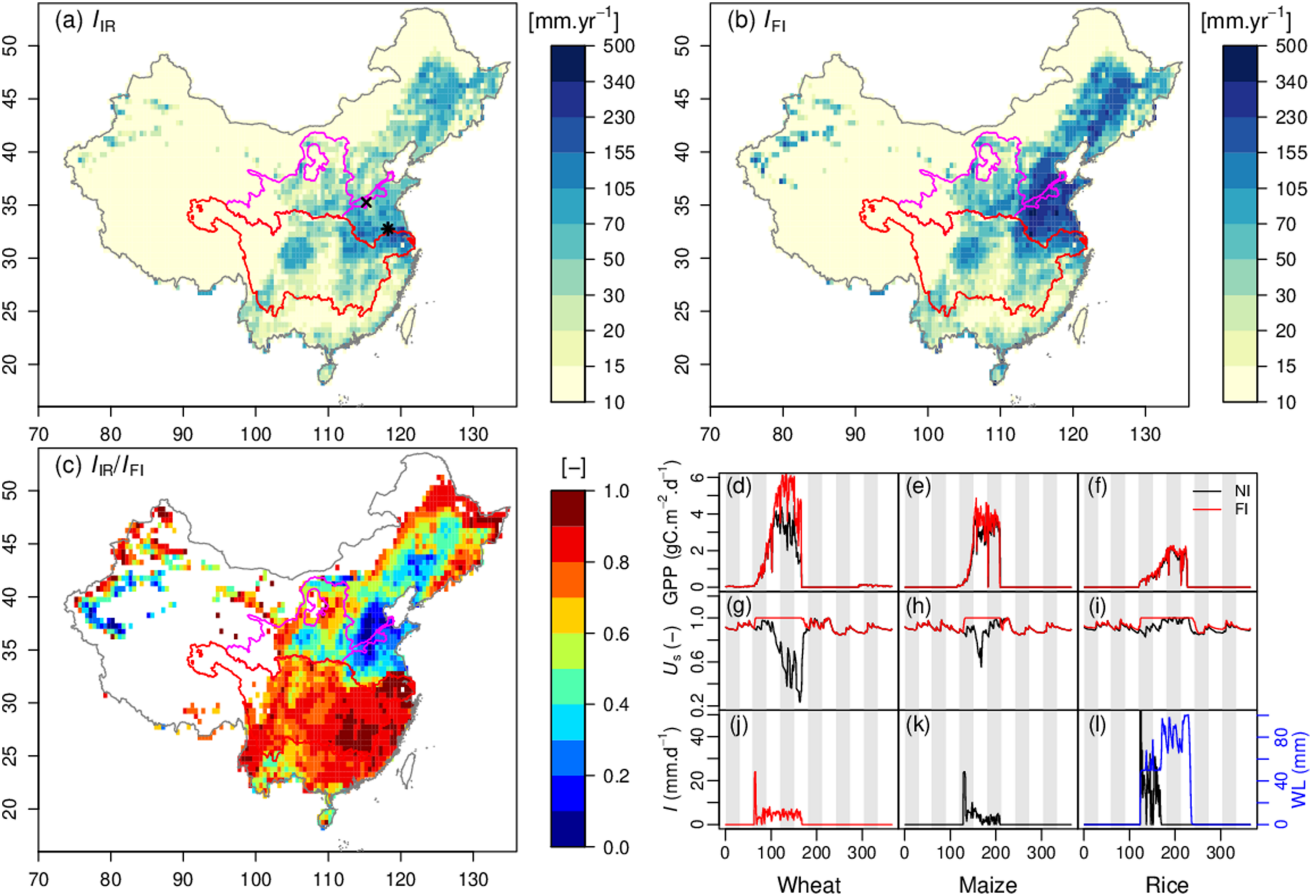
(a) Annual mean I estimated by IR simulation. The color bar is at log ${}_{10}$ scale. (b) Same as (a), but from FI simulation. (c) Ratio of $I_{\text{IR}}$ over $I_{\text{FI}}$. (d)–(l) Performances of the new irrigation module in wheat, maize, and rice simulation. The simulations of wheat and maize are selected from the North China Plain, highlighted as × in Figure 3a. The simulation of rice is from the Yangtze Plain, shown as ∗ in Figure 3a. x axis is day of year, while the white and gray background highlights different months. Dark and red lines represent for NI and FI simulations, respectively, and they are used to show three simulated variables: gross primary production (GPP), hydric stress of plants ( $U_{s}$), and irrigation amount ( I). Blue line represents simulated water level of paddy irrigation in FI simulation.

Figure 3 shows the spatial patterns of simulated irrigation amount ( I, equivalent to $I_{\text{app}}$ in equation (2), the same below) averaged for the period 1982–2014. High I values in Figures 3a and 3b are mainly found in the Northeast China Plain, the North China Plain, the Sichuan Basin, and the Yangtze Plain (definition of those regions in Figure S2). The patterns of $I_{\text{IR}}$ and $I_{\text{FI}}$ are similar, but the magnitude of $I_{\text{FI}}$ is higher than that of $I_{\text{IR}}$ by 97.1% on average, because irrigation application in the FI configuration was not constraint by water supply. The largest differences between $I_{\text{IR}}$ and $I_{\text{FI}}$ occur in the North China Plain, with average value 166.2±84.4 mm ·year ${}^{-1}$. I is lower than 15 mm ·year ${}^{-1}$ in Southern China where rice is the dominant crop. In this wet region, the simulated I of rice paddies is 140 mm ·year ${}^{-1}$, given a rice fraction below 15%. Irrigation mainly occurs from February to July with maximum in May (Figure S3). In the YZRB, monthly $I_{\text{IR}}$ is close to $I_{\text{FI}}$ because there is sufficient surface water supply for irrigation. However, $I_{\text{IR}}$ is much less than $I_{\text{FI}}$ in the YLRB suggesting water stress for irrigation (in absence of renewable groundwater, since we did not simulate this pool). Moreover, high spatial variations of $I_{\text{FI}}$ are found in February, March, and May suggesting large uncertainties of irrigation demand due to climate fluctuations.

The ratio of $I_{\text{IR}}$ to $I_{\text{FI}}$ displayed in Figure 3c reflects water limitation for irrigation in absence of extra supply from groundwater. The largest supply limitations ( $I_{\text{IR}}/I_{\text{FI}}<0.4$) are found in the North China Plain, Loess Plateau, Northeast China Plain, and Northwest China. In these regions, local available freshwater can only meet as low as 20% of the simulated irrigation demand when assuming as in our simulations that all crops are irrigated. In reality, upstream river discharge can be partly used to irrigate croplands, and fossil groundwater is another possible source for irrigation. This is particularly the case of the North China Plain. From the fraction of area equipped for irrigation ( f, Figure 2b), we may find some clues of this water usage as f in the North China Plain is much higher than other regions. According to provincial irrigation statistics, the three provinces located in the North China Plain (Hebei, Henan, and Shandong) used 140 $\times \;10^{8}$ m ${}^{3}\cdot$year ${}^{-1}$ for irrigation, and 65% of the water used comes from deep groundwater (Zheng et al., 2004). This compares well with the estimation ( $1-I_{\text{IR}}/I_{\text{FI}}$, 69%) in our simulations. On the contrary, groundwater resource in the Loess Plateau is limited, and river water is the main source for irrigation, resulting in low f. The averaged I from data reported in the Loess Plateau (Gansu, Shaanxi, Shanxi, and Ningxia) is 59.7 $\times \;10^{8}$ m ${}^{3}\cdot$year ${}^{-1}$ with 35% from groundwater, in comparison with 44% in our simulations.

To illustrate the performances of the crop model with the irrigation module, Figures 3d–3l show daily time series of irrigation‐related variables at sample grid cells (indicated by × and * in Figure 3a) on average in 1990s. The variation of crop Gross Primary Productivity (GPP; Figures 3d–3f) displayed in Figures 3d–3f shows short phenology and growing season of crops. For instance, winter wheat is planted around mid‐October and grows up in the next spring which is consistent with data from FAO (2019) and Li et al. (2005). In the wheat and maize grid cells, GPP is 46% and 12% higher with irrigation, and growing season drought is alleviated. The yield is 45% and 16% higher than without irrigation, respectively. This result coincides with statistics and experiments. For instance, Liu et al. (2007) reported that the yield of irrigated wheat was 76% higher than that of rainfed wheat in Henan province, in which fertilization was taken into account. In several control experiments in the North China Plain, wheat yield can increase from 22% to 67% with irrigation at critical growing stages (Li et al., 2005; Sun et al., 2006; Zhang et al., 2017). Simultaneously, irrigation can increase 19% of maize yield in the North China Plain (Chen et al., 2008; Liu et al., 2017). The positive effect of irrigation is marginal for rice in Southern China where water limitation is not frequent (Figure 3i), consistent with the fact that rice irrigation is not performed for meeting crop water demands but for physiological needs, and avoiding weeds and pests (Bouman et al., 2007). Simulated paddy irrigation consequently has little effect on $U_{s}$, GPP, or yield. Daily irrigation amount ( I) and WL of paddy are shown in Figures 3j–3l. A peak of I is simulated in the first days after planting in order to reach the targeted $U_{s}$. After that, I is close to the daily loss of SM due to ET. For paddy, I can keep the WL at WL ${}_{\xi }$ all the time, and WL exceeds WL ${}_{\xi }$ after rainfall events. Since mid‐June, precipitation is sufficient to maintain the WL over WL ${}_{\xi }$ resulting in near‐zero irrigation (Figure 3l).

At national scale, irrigation increases crop yield substantially (Figure S4). Simulation results show that irrigated crop yield (the FI case) is 24.6%, 32.5%, and 9.2% higher than the rainfed one (the NI case) for wheat, maize, and rice, respectively. Moreover, irrigation reduces the inter‐annual variation of crop yields (coefficients of variation in Figure S4) under climate variation. However, due to the lack of census data separating rainfed and irrigated crop yields, it is hard to evaluate the magnitude of irrigation induced crop yield increase. We did not attempt to compare the trend of yield induced by irrigation to statistical yield data.

### 3.2 Simulated Irrigation Versus Statistical Water Withdrawal

#### 3.2.1 Regional Differences of Irrigation

**Figure 4:**
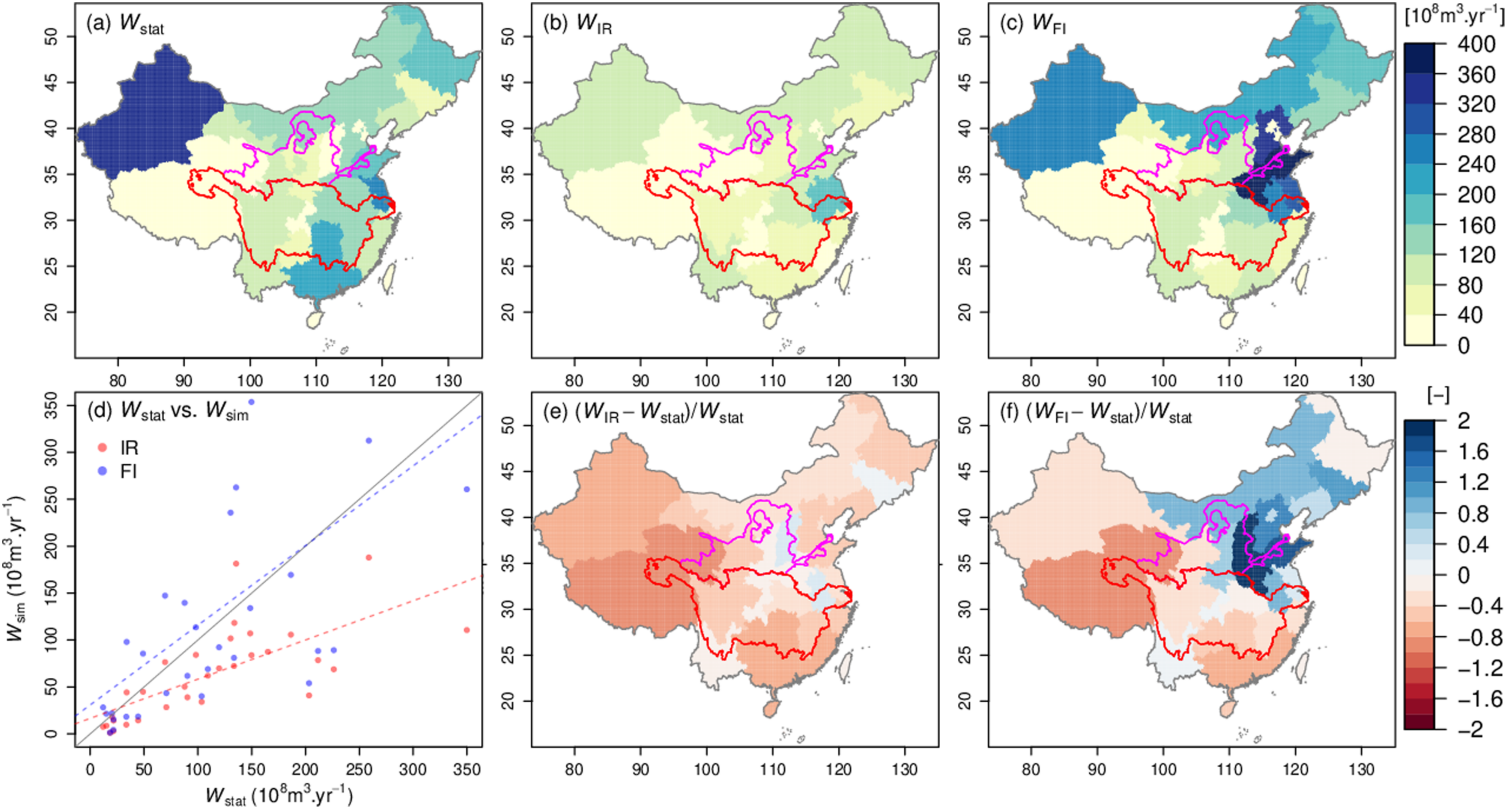
(a) Mean annual statistical W at provincial scale. (b, c) Mean annual simulated W based on IR and FI simulations, respectively. (d) Scatter plot between statistical and simulated W. Points indicate multi‐year averaged provincial W. Dashed lines are linear regression lines, and the solid line is 1:1 line. (e, f) Relative difference between simulated and statistical W.

Figures 4a–4c compare provincial statistics and simulated mean annual W. Note that W is determined by both irrigation intensity and area; that is, Northwest China shows the highest W due to both large irrigation area and intensity. In Figure 4b, the modeled $W_{\text{IR}}$ averaged for each province was higher in the North China Plain, Northwest China, Northeast China, and the Yangtze Plain, which is in rough agreement with $W_{\text{stat}}$( r=0.51; p<0.01), but the magnitude is systematically underestimated (Figures 4c and 4d). The underestimation in the North China Plain is due to the lack of groundwater depletion and water transfer in our simulations. For instance, river water can be transferred for irrigation over long distances, while in ORCHIDEE only grid cells containing river streams are allowed to withdraw water. Thus, the amount of available water resources for areas far from rivers is underestimated, leading to $W_{\text{IR}}<W_{\text{stat}}$. The largest model underestimation is found in Western China, not only due to ignored groundwater which only accounts for 20% of W (Zheng et al., 2004) but also to the lack of glacial runoff in spring (Fang et al., 2018) and to the small fraction of sown area of cereals, accounting for wheat, maize, and rice. For instance, the area with cereals only takes 30% of total sown area in Qinghai (Zheng et al., 2004), implying that irrigation on 70% of sown area with other crops was overlooked in our simulations. The underestimation of $W_{\text{IR}}$ in Southern China may be attributed to the fact that we simulated one growing season (one harvest) per year, while the climate condition allows more than two harvests in that region (Piao et al., 2010; Yan et al., 2014). The fully irrigated irrigation amounts $W_{\text{FI}}$ are in better agreement with $W_{\text{stat}}$ in Western and Northern China but are underestimated in Southern China and overestimated in the North China Plain (Figures 4c and  4f). The FI simulation results regressed against provincial data in Figure 4d show no significant bias across the different provinces (slope =0.85) but a large scatter (relative RMSE, $\sqrt{\frac{\sum_{i=1}^{n}{\left(S_{i}-O_{i}\right)}^{2}}{n}}/Ō$, is equal to 0.89. $S_{i}$ and $O_{i}$ are simulations and observations, respectively. n is the number of samples. Ō is the mean of $O_{i}$), whereas the IR results indicate a linear bias (slope =0.42; relative RMSE =0.67).

#### 3.2.2 Trends of Irrigation

**Figure 5:**
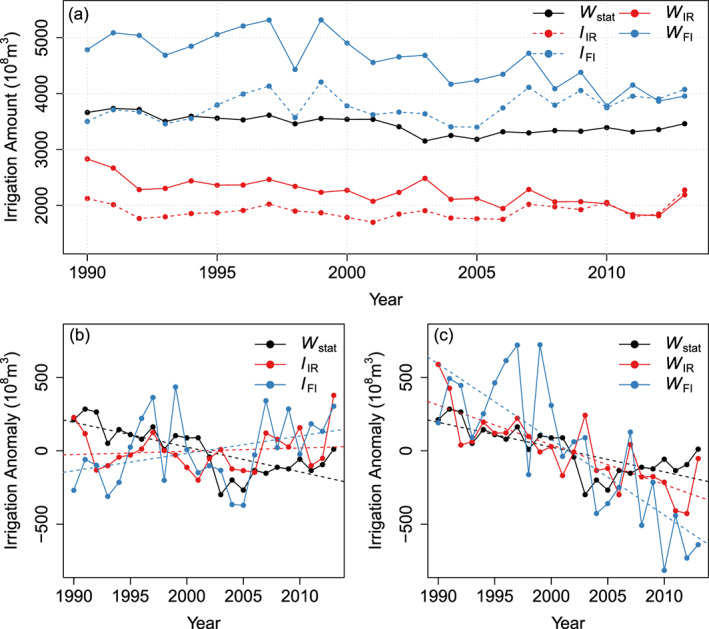
Top panel: Time series of simulated I and W, and statistical W over China. I is the irrigation amount. W is water withdrawal, which is irrigation amount corrected by irrigation efficiency and irrigation fraction (equation (3)). Bottom panel: Comparison of the trends of I and W.

Figure 5a displays the long‐term dynamics of I and W over China. The sum of provincial W ${}_{\text{stat}}$ lies in between simulated W ${}_{\text{IR}}$ and W ${}_{\text{FI}}$, suggesting that our two end‐members simulations comprise a realistic envelop for irrigation withdrawal in reality. The additional sources of glaciers, groundwater and reservoir water not accounted as mentioned above, may contribute to the underestimation of W in the IR simulation. Moreover, anthropogenic factors, such as multicropping, irrigation for other crops (e.g., cash crops), and expansion of irrigation area, may affect $W_{\text{IR}}$ as well. For instance, the total irrigation area increased from 474.0 to 645.4 ( $\times 10^{3}$ km ${}^{2}$) in the period 1990–2014 (Zheng et al., 2004), while only a static f map with an area of 619.0 $\times 10^{3}$ km ${}^{2}$ was used to calculate W. The data in Figure 5b show an increase of modeled I for both IR and FI simulations, but a decrease of $W_{\text{stat}}$. Simulated I reflects irrigation demand increase with climate change in the period of 1982–2014. This climate‐driven positive trend of $I_{\text{FI}}$ is larger than $I_{\text{IR}}$ where local freshwater availability limits the increase of irrigation over time. Nevertheless, in ORCHIDEE, climate change and the physiological effects of rising CO ${}_{2}$ do not explain the decreasing $W_{\text{stat}}$ well over China. According to equation (3), trends in W are not only determined by I but also by trends of f and ϵ. Using GMIA f and the evolution of ϵ over China to convert I into W, the observed decreasing trend of W can be captured (Figure 5c). The trends of $W_{\text{IR}}$ and $W_{\text{FI}}$ were significantly affected by ϵ, which reflects the importance of improvement of water transfer techniques for modeling W trends. In addition, including the effects of f and ϵ are not only important to model W trends, but they also improve the spatial patterns of irrigation compared with $W_{\text{stat}}$ (Figure S6).

### 3.3 Irrigation Impacts on Water Cycles and Surface Energy Budget

**Figure 6:**
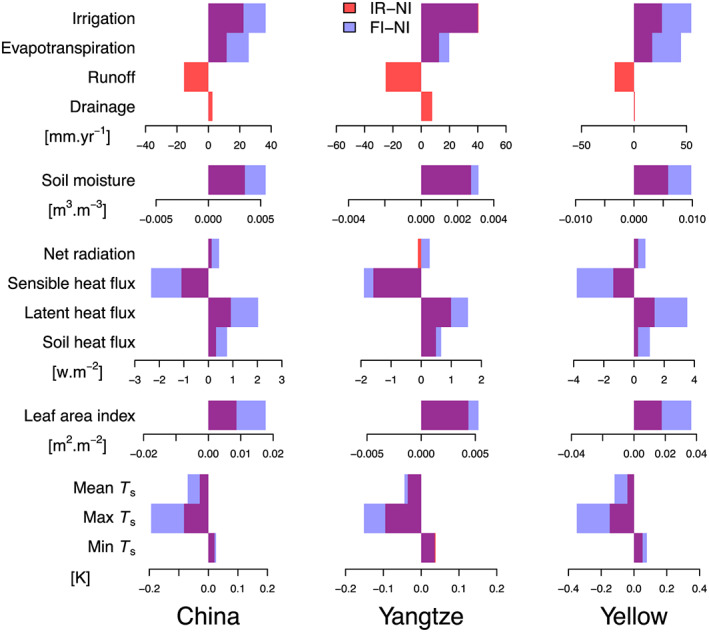
Impacts of irrigation on water budgets and surface energy balances in the simulations. Histograms represent the differences of specific value between IR (or FI) and NI simulations. $T_{s}$ is surface temperature. Three columns are for China, YZRB, and YLRB, respectively. As FI does not account for water balances, there is no plot of FI effects on runoff and drainage.

Figure 6 presents the impacts of irrigation on the water and surface energy fluxes. Averaged over China (left panel of Figure 6), I has a positive impact on ET, SM, and deep drainage, but a negative one on runoff. Significant ET increase is found in the YLRB from March to July by as much as 33.6% (Figure S7). However, such difference is less than the uncertainties given by the spread of different ET products, implying difficulty to validate the impact of irrigation on ET by current ET data sets. Irrigation also increases net radiation ( $R_{n}$), because it promotes LAI by reducing water stress and consequently decreases surface albedo. Last, I increases expectedly latent heat flux and decreases sensible heat flux leading to a slight decrease of surface temperature ( $T_{s}$) over irrigated crop tiles. The irrigation induced evaporative cooling mainly affects maximum $T_{s}$, while minimum $T_{s}$ values at nighttime are not significantly influenced. At the basin scale, surface runoff in the YZRB is significantly reduced due to paddies that keep water on the cropland and decrease runoff yield. In the YZRB, $R_{n}$ is slightly decreased in IR simulation due to a small increase of min $T_{s}$, which increases longwave radiation emitted from the surface. The FI simulation leads to a larger ET increase than the IR one across China, but this effect is more pronounced in the YLRB than the YZRB. Even though the modeled $I_{\text{IR}}$ is smaller in the YLRB (20.9 mm ·year ${}^{-1}$) than that in the YZRB (32.0 mm ·year ${}^{-1}$). However, the impacts of $I_{\text{IR}}$ on climatic variables in YLRB are more significant than that in the YZRB, suggesting more positive impacts of I on ET in the dryer YLRB where irrigation alleviates water stress.

Year‐to‐year anomalies of I may be expected to correlate negatively with P. Less P in a dryer year should lead to increased I to compensate for the plant water demand. This is the case in the FI simulation results (Figure S8b), in which irrigation is not limited by water availability. However, when water limitation is included for the IR simulation, a positive correlation is found over Northern China (Figure S8a). This is because in this region, I anomalies are controlled by the supply from freshwater determined by P. For example, if P is less, water supply for irrigation is less, meaning less I, and vice versa. We found that there is a threshold of annual P(1,000 mm ·year ${}^{-1}$) below which appears a positive correlation between I and P anomalies (Figure S9). Such correlation may additionally depend on P seasonality and f.

A key impact of irrigation is that it allows annual ET to exceed P. Figures S8c and S8d display the average P−ET from IR and FI simulations. Negative values occur in the Northern China with similar patterns to I. The averaged P−ET in the North China Plain is approximately −200 mm ·year ${}^{-1}$. In the observation‐based products, there are also negative values of P−ET(Figure S10) in the SEBS, FLUXCOM, and PKU gridded ET products but not in the GLEAM product, for this region. The GLEAM ET model assimilates CCI SM values (Dorigo et al., 2015; Martens et al., 2017) but is still constrained by annual local input from P thus discarding added irrigation water (section 2.2.3), explaining why it does not account for a negative water balance.

### 3.4 Effects of Irrigation on Total Water Storage Trends

**Figure 7:**
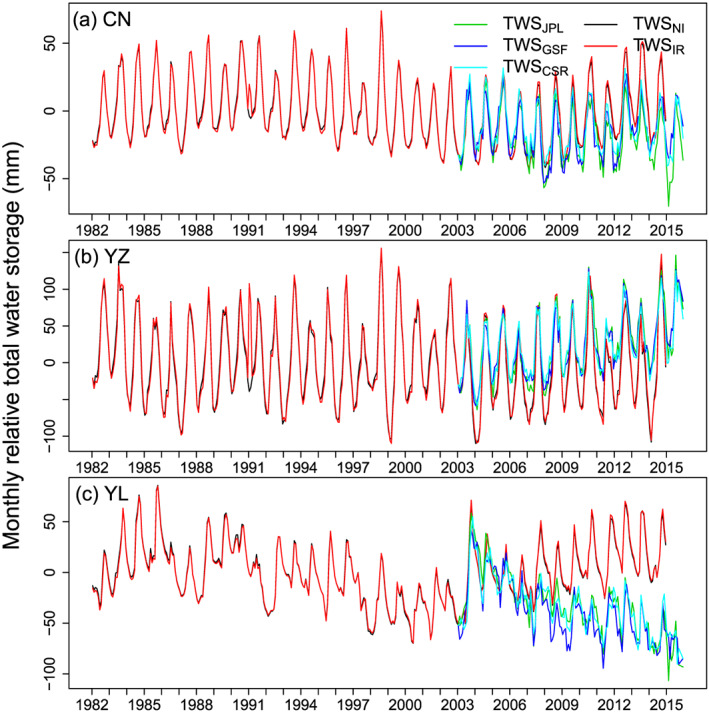
Time series of simulated and GRACE‐based TWS in different regions. To better illustrate the comparison, we let the start value of GRACE‐based TWS equal to the value of TWS ${}_{\text{IR}}$ at the same time step.

**Figure 8:**
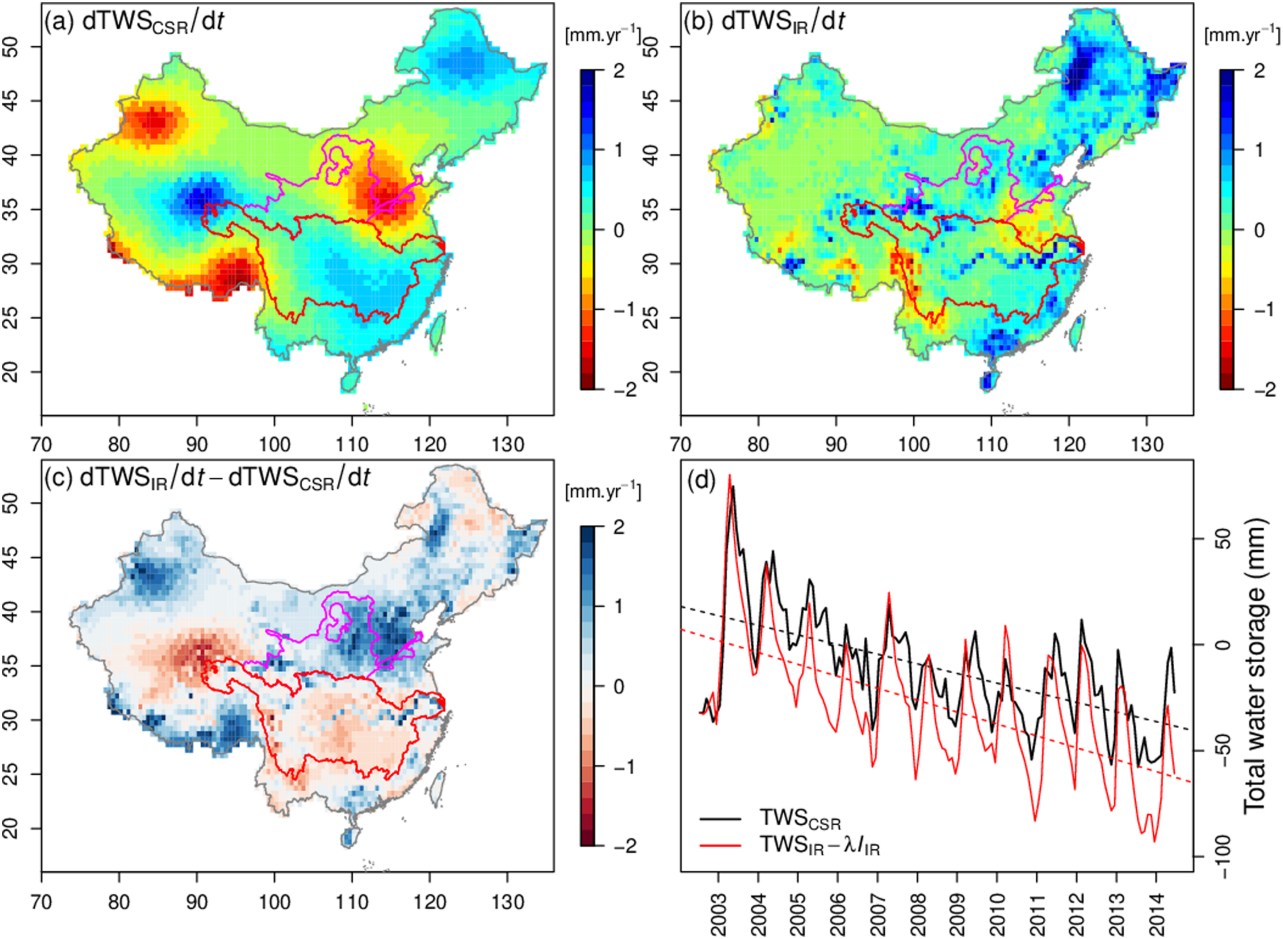
(a) and (b) Trends of observed(CSR) and simulated (IR) TWS at each grid cell in period 2003‐2014, respectively. (c) The difference of trends between TWS ${}_{\text{IR}}$ and TWS ${}_{\text{CSR}}$. (d) The time series of TWS ${}_{\text{CSR}}$ and corrected TWS ${}_{\text{IR}}$. λ (0.442) represents the ratio of groundwater withdrawal over surface water withdrawal retrieved from the Year Book of China.

Figure 7 shows time series of simulated TWS compared to GRACE estimations. The TWS ${}_{\text{NI}}$ and TWS ${}_{\text{IR}}$ model results almost overlay, implying that I has no significant effect to TWS if only surface water resources are taken into account. In our model where irrigation is just a transfer of water from streams to soils, simulated TWS matches the seasonality of GRACE TWS well. This indicates that the first‐order seasonality of TWS is driven by the seasonality of P−ET. Nevertheless, a seasonal amplitude bias is observed, with an underestimated minimum TWS in the model in late spring over the YZRB (Figure S11b), which is probably due to the lack of water storage regulation by artificial reservoirs in the model. To reduce peaks of floods and provide water for irrigation, reservoirs in the YZRB accumulate water after the flooding season and release it later for irrigation in spring. For instance, the Three Gorges Dam (TGD) starts to recharge water from early September to mid‐October and reduces its storage from January to May (blue and red areas in Figure S11b). When the contribution of this single large dam to the TWS variation in YZRB is taken into account (cyan line in Figure S11b), the RSME of TWS ${}_{\text{IR}}$ decreased by 7.9% (from 42.0 to 38.7 mm). In fact, the total regulation capacity of artificial reservoirs in the YZRB is 770 $\times 10^{8}$ m ${}^{3}$, which is equivalent to 42.8 mm, close to the misfit between simulated and GRACE TWS. Further, reservoir regulation varies between years, whereas we applied here a monthly TGD storage correction in 2016 to the period of 2003–2014, and the operations of reservoirs are not synchronous, being driven by irrigation but also industry and drinking water demand and hydropower. Spatial patterns of the trends of GRACE TWS (here the TWS ${}_{\text{CSR}}$ product) and TWS ${}_{\text{IR}}$ are displayed in Figure 8. The trend of TWS ${}_{\text{IR}}$ is driven by the trend of P, which has the same patterns (Figure S12). In the regions with significant groundwater depletion (the North China Plain and Northwest China, Rodell et al., 2018), TWS ${}_{\text{IR}}$ cannot capture the decreasing trends observed by GRACE. To check the relationship between observed TWS and groundwater usage in China, we collected data on water withdrawal records in provinces that mainly locate in the YLRB during the period 2003–2014 (Table S3). These data show that groundwater withdrawal represents a fraction λ=0.442 of total water withdrawal. Removing from modeled TWS ${}_{\text{IR}}$, a groundwater depletion term of $\lambda I_{\text{IR}}$ brings the simulated TWS trend ( −5.4 mm ·year ${}^{-1}$) in close agreement with GRACE observations ( −5.36 mm ·year ${}^{-1}$). This result confirms that groundwater depletion can explain the trend of TWS in regions where stream water is not sufficient.

### 3.5 Effects of Land Use Change on the Trend of Irrigation

**Figure 9:**
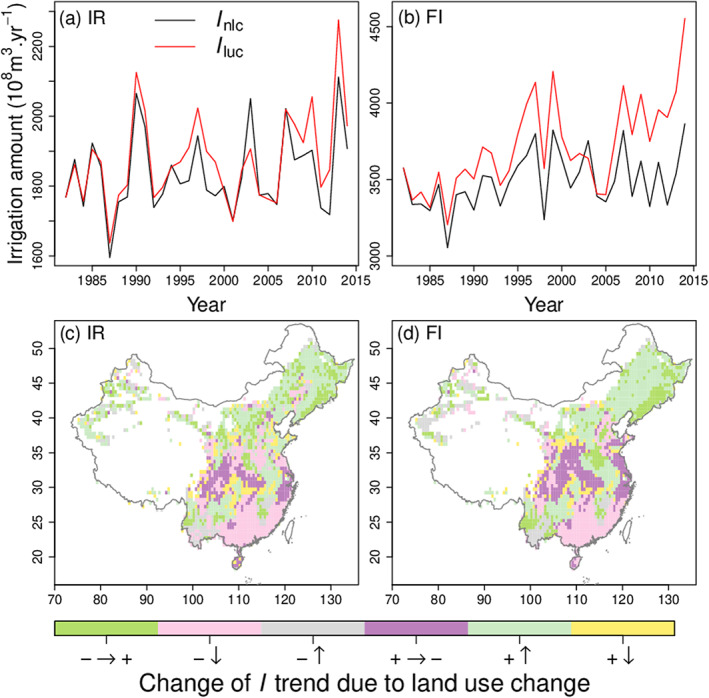
(a) Time series of annual irrigation amount ( I) over China based on the IR simulation. $I_{\text{luc}}$/ $I_{\text{nlc}}$ indicates simulated irrigation amount with/without land use change. (b) The same as (a), but in the case of FI. (c) How land use change affects the trend of simulated irrigation amount based on the IR simulation. + and − indicate increasing and decreasing trend, respectively. ↑ and ↓ indicate that the I trend increased or decreased after considering land use change, respectively. (d) The same as (c), but in the case of FI.

Cropland area increased over the North China Plain, Northeast China, and East of the Loess Plateau from 1982 to 2014 (Figure S13c) but decreased in the rest of China. It is of interest to analyze how land use change (LUC) can affect the trend of simulated I in our model. Figures 9a and 9b compare the time series of I with and without LUC for IR and FI, respectively. Constrained by water availability in the IR case, the total I is slightly promoted by LUC. In the Yangtze Plain, the cropland area decrease has offset the rising I due to climate change (increasing ET ${}_{p}$; Figure S5a). In Northeast China, climate change enhanced I, and expanding cropland areas further increased the irrigation requirement. Without limitation by water in the FI case, LUC significantly amplifies the total I (Figure 9b), especially in the North China Plain and the Loess Plateau, where cropland expanded (Figure S13c). Figures 9c and 9d show the change of I trend due to LUC. Cropland decrease in Southern China (e.g., the Sichuan Basin, the Yangtze Plain, and the coastal regions at Southeast China) reduced I. In YLRB, the slightly increase of cropland area combined with climate tends to increase I. Thus, how to optimize agricultural water management in YLRB is a crucial question for sustainable development. The cropland expansion in the Northeast China Plain increased I as well. However, there is no significant drop of TWS (Figure 8a) in the last decade from LUC.

## 4 Discussion

### 4.1 Irrigation Module and Perspectives

Compared to the earlier version of ORCHIDEE used for hydrological modeling (Guimberteau et al., 2012), the current irrigation module is now capable of considering the impacts of several techniques on irrigation practices. Three irrigation techniques are considered, and irrigation demand depends on the specific technique (equation (1)). Independent energy balance is estimated for each soil tiles, which precisely capture the interactions between crops and local climate. Moreover, three scenarios are developed for different irrigation scenarios. One includes water balance, in which irrigation will be limited by renewable water resources. It is used to investigate the effects of irrigation practices on hydrological cycle. Another scenario fulfills irrigation demand excluding water balance. The aim is to estimate the potential yield of crops without water limitation. Moreover, it can help to understand irrigation variation where groundwater is widely used. Another feature is paddy irrigation, which is a popular treatment for paddy rice particularly in major rice producing countries over Asia (Salmon et al., 2015). Although the water level of paddy does not influence photosynthesis, it accounts for compulsory irrigation in some wet regions where transpiration is not often constrained by soil moisture.

One key question for irrigation models is how to assess irrigation demand. Nazemi and Wheater (2015a) compared irrigation modules in numerous hydrological and land surface models and showed that deficits between potential/targeted and actual ET, TR, and SM were commonly used to estimate irrigation demand. Theoretically, the net irrigation demand should be the gap between crop potential and actual TR, which maximizes crop photosynthesis without water stress. However, it is difficult to reach this target mainly due to limitation of irrigation facilities. For instance, flood irrigation is still widely applied in developing countries. Farmers perhaps will not precisely know the TR deficit and apply more irrigation than needed. Thus, SM deficit is more realistic than the others. In our simulations, the targeted SM is saturation in order to estimate the upper limit of irrigation demand. It is different from other hydrological studies using (a percentage of) field capacity as irrigation target (Hanasaki et al., 2008; Wada et al., 2016). However, as deep drainage was turned off in our study, simulated I is equivalent to evaporation deficit at long‐term scale.

However, the new irrigation module is not yet sufficient to fully represent the complexity of irrigation practice. First, not all water resources are taken into account. For instance, the lack of simulating nonrenewable groundwater leads to large uncertainties in estimating I in arid and semiarid regions. Although the FI simulation and statistical data can help us to understand the irrigation demand and TWS trend in arid and semiarid regions, linkages between surface water and groundwater are not negligible for future projection. Moreover, water transfer is not yet included in the model, which may result in underestimating acquisition ability of water for irrigation. Second, the information on irrigation is not enough. Irrigation fraction ( f), as one of the important irrigation factors, is not well demonstrated. Large mismatches exist between different f maps (Liu et al., 2018). Furthermore, static f map is not sufficient to represent the fast growth of irrigated area over China in the last three decades, which can strongly influence I. Another important factor is irrigation efficiency ( ϵ, equation (3)), which was found to partly explain the trend of W in China (section 3.2). However, such statistical ϵ only available at national level cannot reproduce the spatial variations of irrigation techniques development.

### 4.2 Irrigation Water Withdrawal in China

The modeled amounts of irrigation withdrawal $W_{\text{IR}}$ and $W_{\text{FI}}$ bracket the provincial data $W_{\text{stat}}$ with relative differences ranging between 11.4% and 50.0%. Systematically, underestimation of W is found in Northwest and South China, including Loess Plateau, North China Plain, and Northeast China Plain, which are under water scarcity. For example, groundwater W accounts for 44–83% of total W in related provinces in 2004 (Zheng et al., 2004). This report coincides with our results that simulated surface water withdrawal ( $W_{\text{IR}}$) can be as low as 53.0% of $W_{\text{stat}}$. Due to the extreme assumption of the FI simulation, $W_{\text{FI}}$ overestimates W in these water limited regions. The spatial correlation can test whether the model can capture patterns of irrigation demand across different climatic regimes. A fair agreement is found between I and $W_{\text{stat}}$ (Figure S6). Moreover, the spatial correlation can be significantly improved by including information of f and ϵ, indicating that anthropogenic factors cannot be negligible in estimating irrigation demands and water withdrawal.

On average across China, W decreased by more than 200 $\times 10^{8}$ m ${}^{3}$ in the period of 1982–2014. However, this trend is not completely reproduced in our simulated I ( $I_{\text{IR}}$ and $I_{\text{FI}}$ increased 150.4 and 573.0 $\times 10^{8}$ m ${}^{3}$, respectively), which only reflect the impacts of climate change (including the physiological effect of CO ${}_{2}$) and land use change on W (Fischer et al., 2007; Hanasaki et al., 2013; Konzmann et al., 2013; Leng, Huang, et al., 2015). According to our model results, the irrigation demand, that is, $I_{\text{IR}}$ and $I_{\text{FI}}$, increased during the simulated period. Thus, the improvement of irrigation efficiency ( ϵ) is the only factor that can explain the decrease of W based on our current diagnosis (equation (3)). In fact, the change of irrigation fraction ( f) certainly can affect W trends as well. However, there are no precise gridded data available to describe trends of f in China. Note that f can be influenced by ϵ as well. The increase of ϵ implies a reduction of W over time. However, W probably will not decrease but further used to extend f(Grafton et al., 2018; Ward & Pulido‐Velazquez, 2008). Thus, the relationship between ϵ and f should be clearly demonstrated to project future W under climate change. The trend of W is influenced by land use change as well. Our results demonstrated that the decrease of cropland area has a negative impact on I in Southern China. Meanwhile, cropland area expansion in the North China and Northeast China Plains promoted I.

The variation of TWS is strongly influenced by human activities (Pokhrel et al., 2012; Rodell et al., 2018). In YZRB, simulated TWS overestimated the seasonal variation of TWS, which may be due to the joint regulation of artificial reservoirs. In the YLRB, groundwater depletion leads to significant TWS decline. However, simulated TWS showed an opposite trend as the nonrenewable groundwater dynamics is missing in the model, which is a limitation existing in numerous hydrological and land surface models (Scanlon et al., 2018). Both cases call our attention to the human‐induced water storage change, which should be included in future model development to provide reliable water resources projection under anthropogenic factors and climate change.

The land use change is another key factor for estimating irrigation change (Tong et al., 2003). We found that cropland area decreased by as much as 1.5% per year in Southern China in the last three decades (Figure S13), which compensated for the increase of irrigation demand due to climate change (Figure S5). While in the North China Plain and the Northeast China Plain, the expansion of cropland significantly increased the irrigation demand. However, the impacts of land use change on the water resources amount were not taken into account. For instance, Zhao et al. (2016) found that the expansion of woodland and grassland after the abandonment of cropland reduced blue and green water flows by promoting ET in a sub‐basin of YLRB, which in turn decreased surface water resources for irrigation. In addition, the change of sown crops can vary the seasonality of irrigation demand and water cycle as well (Luan et al., 2018). These mechanisms should be considered in future studies to provide a comprehensive assessment of land use change.

### 4.3 Irrigation and Local Climate

The interactions between irrigation and climate are complex. Climate determines irrigation, which in turn affects local climate through atmospheric feedbacks (Guimberteau et al., 2012; Sacks et al., 2009; Thiery et al., 2017). In our model which does not include any atmospheric coupling effects, the covariation between I and P can be positive or negative, depending upon background water availability (Figure S8). However, this negative relationship between I and P can be weakened if external water resources are used such as groundwater, and detailed time series of irrigation amount would be needed to evaluate this model result.

Surface cooling by irrigation was investigated by numerous studies (Chen & Jeong, 2018; Kang & Eltahir, 2018; Sacks et al., 2009; Shah et al., 2019; Thiery et al., 2017). Sacks et al. (2009) demonstrated that irrigation can cool the surface by −0.5 K in Southeast Asia over year, whereas Thiery et al. (2017) found that the cooling effect is −0.10 K. Our study find that irrigation can decrease $T_{s}$ by 0.05 and 0.13 K in YZRB and YLRB, respectively. The maximum $T_{s}$ decrease is found in the North China Plain with −0.8 K. In fact, this is conjunct results of decreasing max $T_{s}$ and increasing min $T_{s}$, which are consistent with Chen and Jeong (2018) but with different magnitudes. Nevertheless, both studies confirmed that irrigation can reduce temperature amplitude between day and night.

Surface cooling is coupled with ET and TR changes (Seneviratne et al., 2010), and our model shows a strong sensitivity of TR to cooling. In our offline simulations, this sensitivity is probably overestimated, given the lack of atmospheric coupling processes and the single source feature of our representation of the energy budget (the whole canopy is represented as a single source, the air temperature inside the canopy not being resolved). In a coupled land‐atmosphere model, irrigation induced evaporative cooling will induce an air temperature decrease and a near surface relative humidity increase that should reduce transpiration and attenuate evaporative cooling of $T_{s}$.

## 5 Conclusions

The irrigation module presented in this study and coupled to ORCHIDEE‐CROP has the advantage to compute irrigation demand and application for specific irrigated crop soil tiles in each grid cell, allowing mass closure and avoiding to give irrigation water to other vegetation types. Irrigation demand calculation can be chosen for three different irrigation techniques, including paddy irrigation for rice.

The performance of the model was evaluated for the spatial patterns of irrigation withdrawal across different provinces, with both freshwater limited (IR) and full irrigation (FI) simulations showing a spatial correlation of ≈0.68 with independent withdrawal census data. The correlation is larger when irrigation withdrawal in the model includes a realistic irrigated fraction and efficiency. This means that beyond the simulation of plant water demand, irrigation efficiency is important to represent in land surface models to match observed withdrawal. Province level actual withdrawals ( $3451.4\pm 160.5\times 10^{8}$ m ${}^{3}$) lie in between the model results for freshwater limited irrigation ( $2242.9\pm 240.8^{8}$ m ${}^{3}$) and those with full irrigation meeting all the plant demand ( $4596.2\pm 457.6\times 10^{8}$ m ${}^{3}$). On one hand, the amount of irrigation withdrawal could be underestimated in our model because we ignored the consumption for other crops than wheat, maize, and rice and multiple cropping. On the other hand, it can also be overestimated in regions where dams supply additional water and where groundwater is used. In addition, we showed that land use change can substantially influence the trend of irrigation amount with the conjunct impact of climate change.

Regarding the water and energy budgets, irrigation was found to reinforce evapotranspiration (by 11.6 and 25.7 mm in the cases of IR and FI, respectively). It cooled and warmed the land surface during daytime and nighttime, respectively, leading to a decrease of mean surface temperature ( −0.03 K for NI and −0.07 K for FI) over China. We admit that the irrigation induced surface cooling could be overestimated in our simulations where the atmospheric vapor pressure deficit is prescribed.

Variation of simulated total water storage (TWS) is coherent with the GRACE satellite data over China ( r=0.87; p<0.001) from 2003 to 2014. However, the regulation of artificial reservoirs strongly affects the seasonal variation of TWS in the YZRB, and the decreasing trend of TWS in the YLRB ( −5.36 mm ·year ${}^{-1}$) is not captured in our simulation due to lack of groundwater simulation. However, after corrected by groundwater depletion inferred from simulated $W_{\text{IR}}$ and census data, we successfully reproduced the decreasing trend of TWS in the YLRB ( −5.4 mm ·year ${}^{-1}$). Thus, it is crucial to include dams management and groundwater resources in future simulations.

## Supporting information



Supporting Information S1
