# Supplementary material for: Improvement of the Irrigation Scheme in the ORCHIDEE Land Surface Model and Impacts of Irrigation on Regional Water Budgets Over China
Source: J Adv Model Earth Syst. 2020 Apr 24;12(4):e2019MS001770. doi: 10.1029/2019MS001770 (PMC7375161; doi:10.1029/2019MS001770)
Supplement: Supplementary file 1 — Supporting Information S1 [file JAME-12-e2019MS001770-s001.pdf]

Supporting information for “Improvement of the irrigation scheme in the ORCHIDEE land surface model and impacts of irrigation on regional water budgets over China”

Z. Yin[1,2,*], X. H. Wang[1,2,3,*], C. Otté[1], F. Zhou[3], M. Guimberteau[1,4], S. S. Peng[3], S. L. Piao[3], L. Li[2], Y. Bo[3], X. L. Chen[5,6], H. Kim[7], P. Ciais[1]

[1]Laboratoire des Sciences du Climat et de l'Environnement, CNRS-CEA-UVSQ, Gif-sur-Yvette, France [2]Laboratoire de Météorologie Dynamique, UPMC/CNRS, IPSL, Paris 75005, France [3]Sino-French Institute for Earth System Science, College of Urban and Environmental Sciences, Peking University, Beijing 100871, China [4]UMR 7619 METIS, Sorbonne Universités, UPMC, CNRS, EPHE, 4 place Jussieu, Paris 75005, France [5]Key Laboratory of Tibetan Environment Changes and Land Surface Processes, Institute of Tibetan Plateau Research, Chinese Academy of Sciences, Beijing, China [6]CAS Center for Excellence in Tibetan Plateau Earth Sciences, Beijing, China [7]Institute of Industrial Science, The University of Tokyo, Tokyo, Japan

*These authors contributed equally to this work

Corresponding author: Z. Yin, Laboratoire de Météorologie Dynamique, UPMC/CNRS, IPSL, Paris 75005, France. (vyin@lsce.ipsl.fr); X. H. Wang, Sino-French Institute for Earth System Science, College of Urban and Environmental Sciences, Peking University, Beijing 100871, China. (xuhui.wang@lsce.ipsl.fr)

Contents of this file

1. Figures S1 to S13

2. Tables S1 to S3

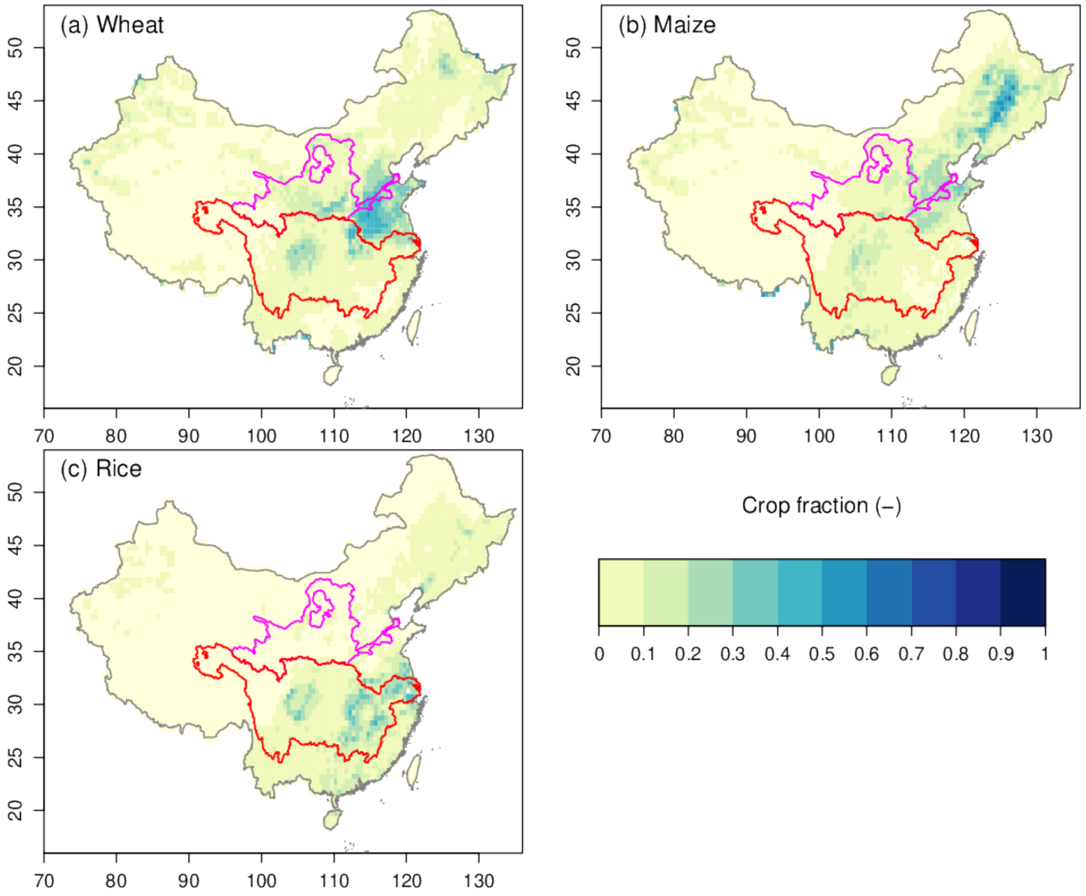

Figure S1. Multi-year averaged crop fractions based on the 15-PFT map.

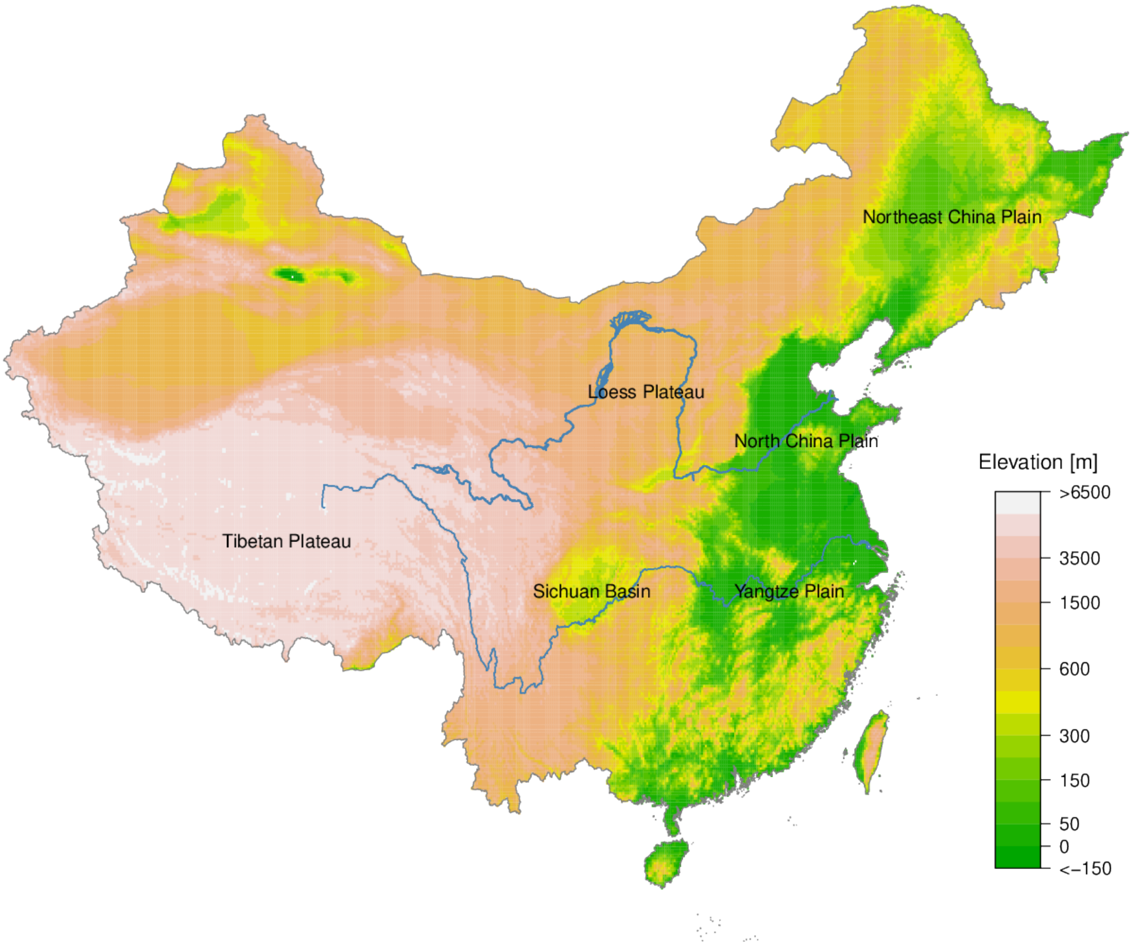

Figure S2. Regions of interest mentioned in this study. The Digital Elevation Model (DEM) is based on the 2000 Shuttle Radar Topography Mission (SRTM; `http://glcf.umd.edu/data/srtm/`).

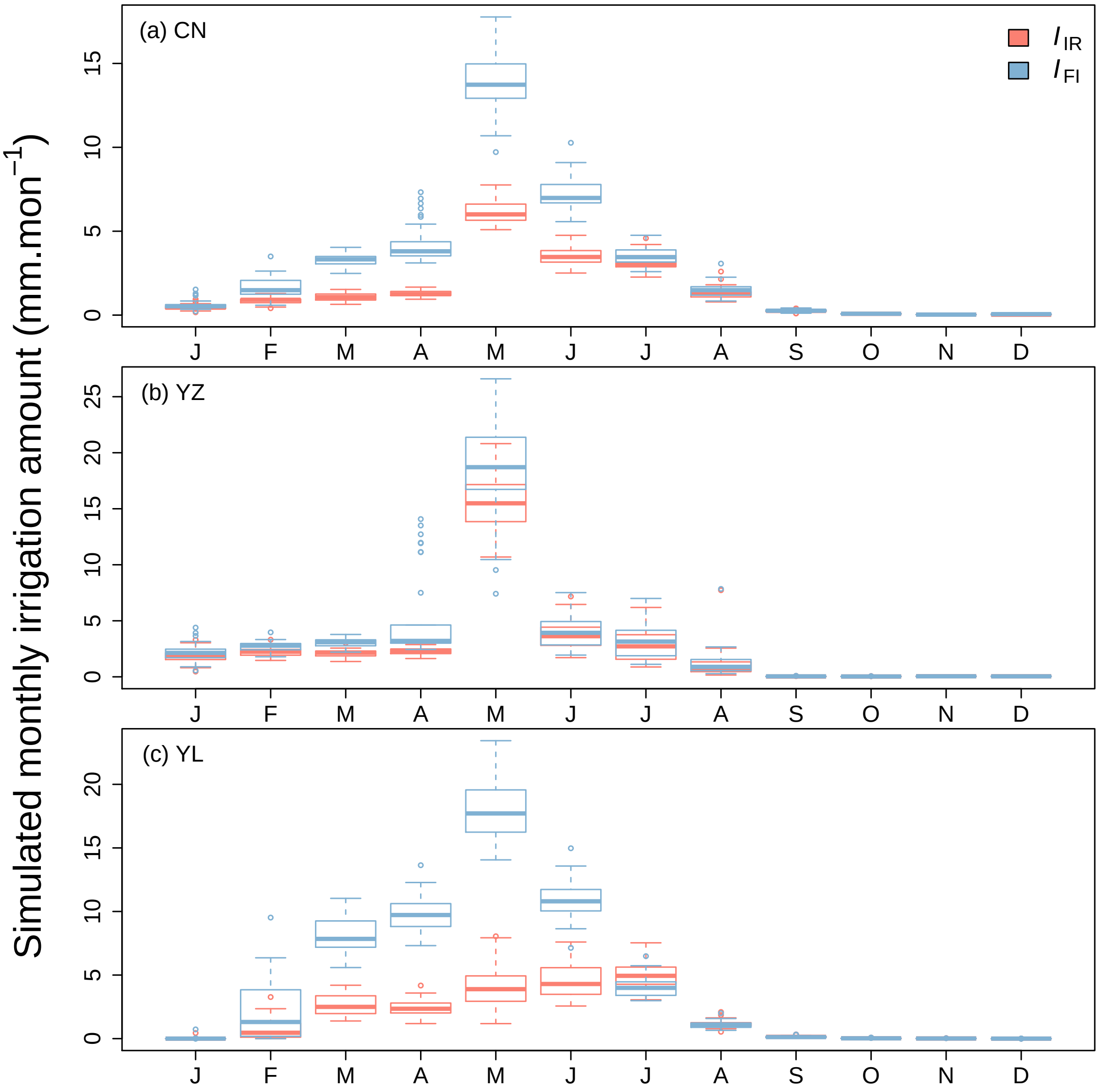

Figure S3. Simulated monthly irrigation amount in China, Yangtze River Basin, and Yellow River Basin. Red and blue indicates NI and FI simulations, resperespectively. Samples in the box plots are spatial averaged irrigation amounts in specific month of different years from 1982 to 2014.

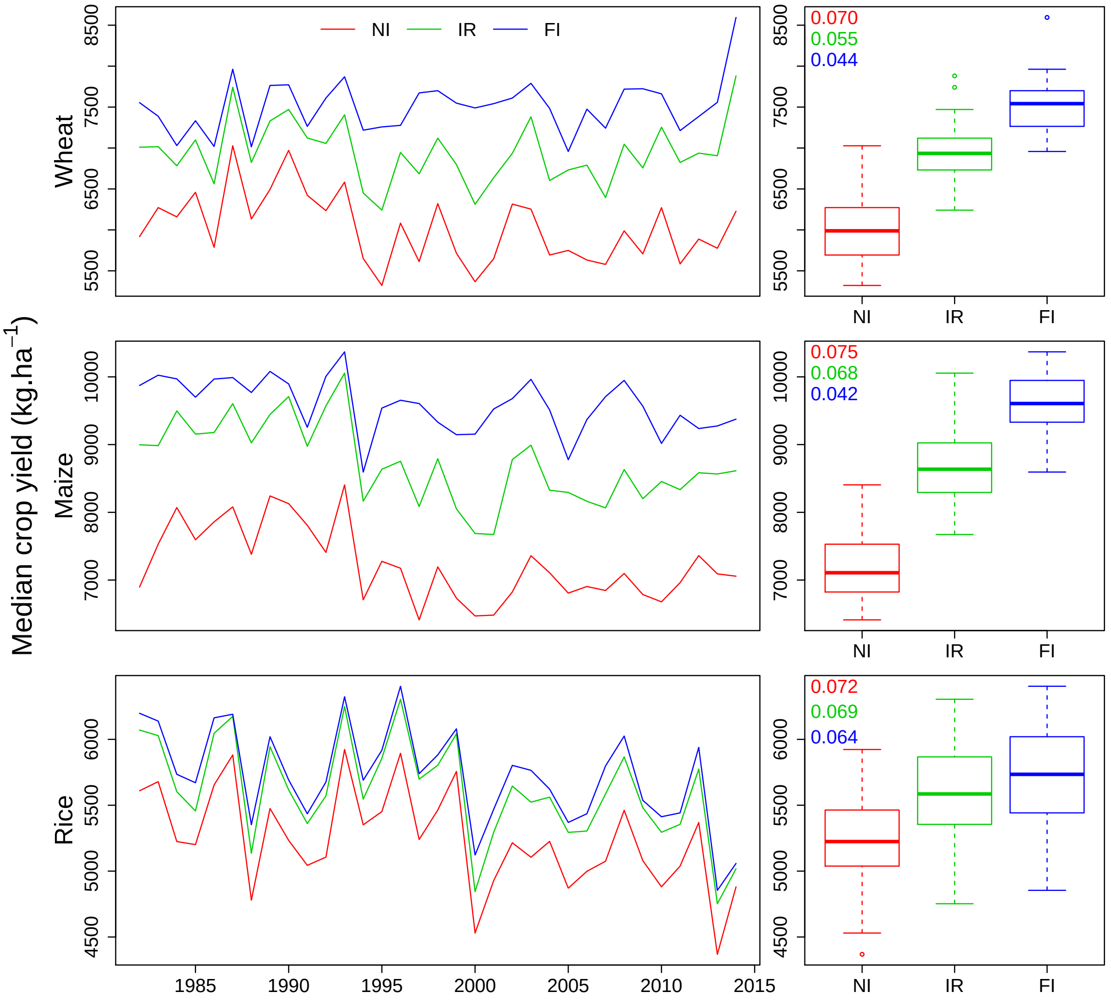

Figure S4. Left panel: Time series of median values of simulated crop yields over China from 1982 to 2014. Colors indicate different simulations. Right panel: box plots of simulated crop yields from all grid cells over China during the simulation period. The three rows from top to bottom are for wheat, maize, and rice, respectively. Three values at the top left corner are coefficients of variation (ratio of standard deviation over mean) of the annual median crop yields of corresponding crops (left panel).

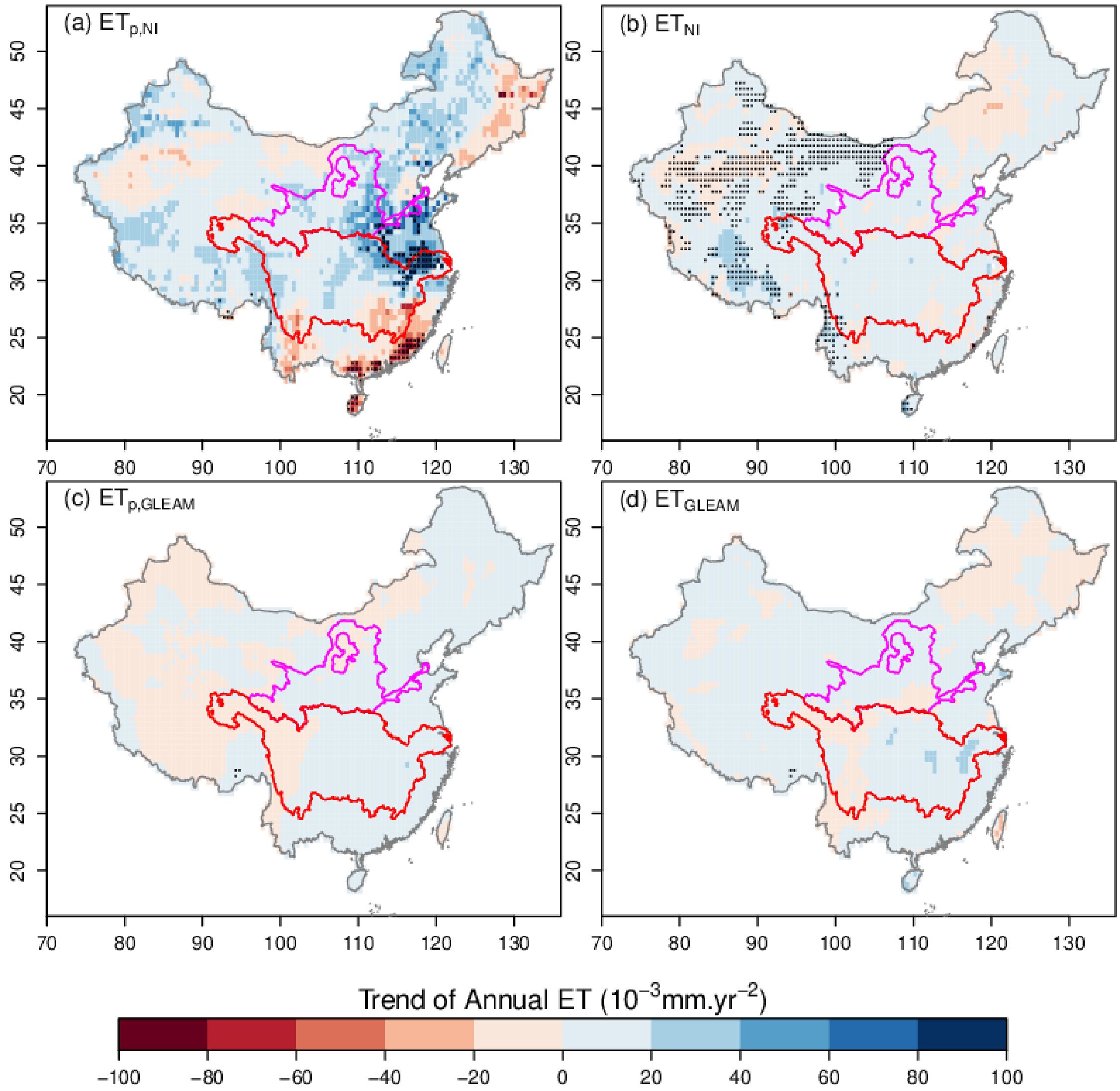

Figure S5. Trends of potential and actual ET based on NI simulation and GLEAM. Dark points indicate the trend is significant ($p < 0.05$ according to Mann-Kendall test.)

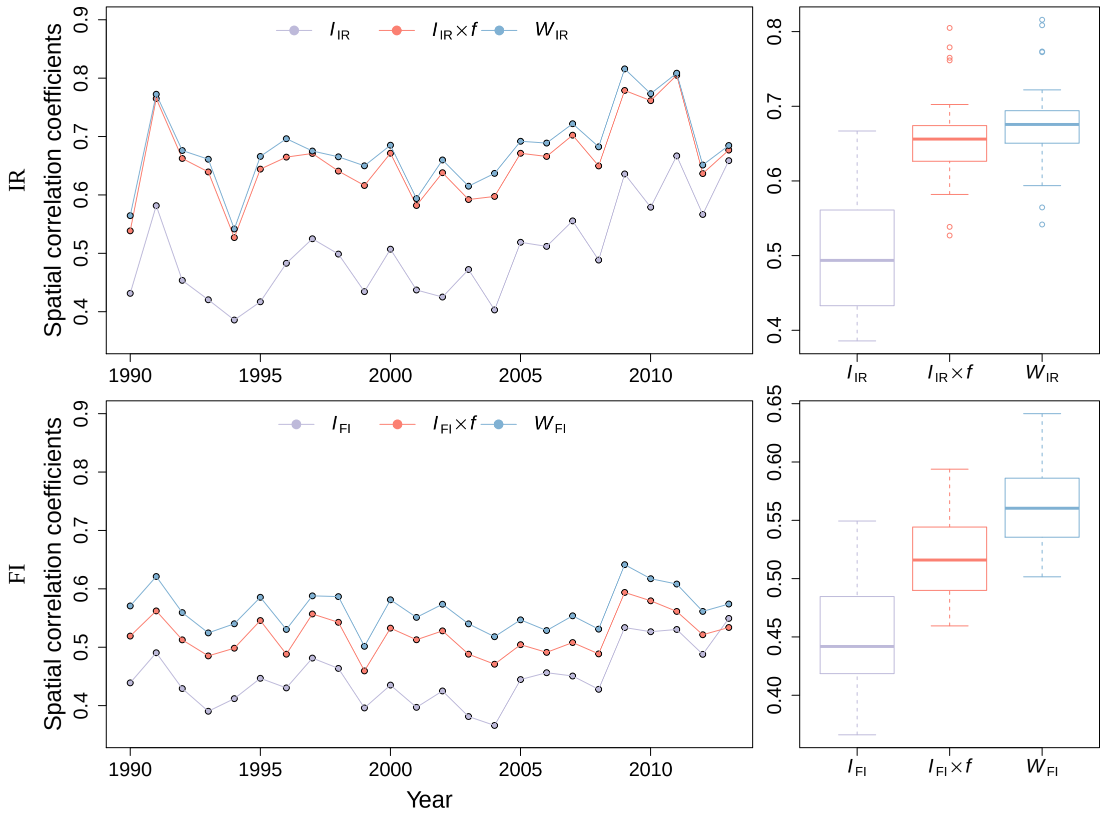

Figure S6. Left panel: time series of spatial correlation coefficients between simulated variables and statistical W. f is irrigation fraction (Fig. 2(b)). Circles with black border represent significant correlation ($p < 0.05$) according to Mann-Kendall test. Right panel: bar plot of spatial correlation coefficients corresponding to the left penal. The difference between every two terms are significant ($p < 0.05$) according to t-test.

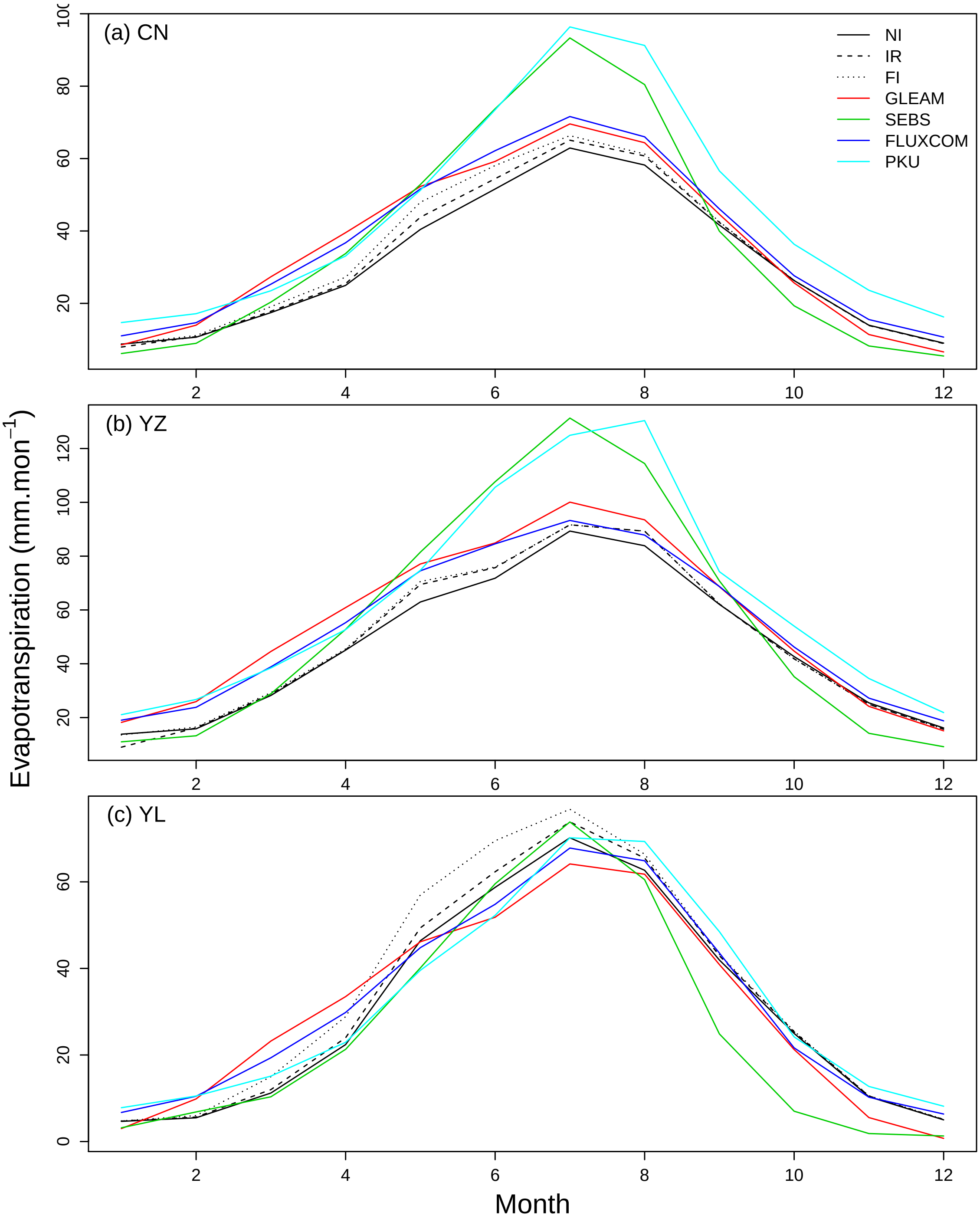

Figure S7. Seasonal cycles of ET from simulations and different ET products in different regions. (a), (b), and (c) are for China, Yangtze River Basin, and Yellow River Basin, respectively. Simulated ET can be distinguished by different styles of dark lines. ET products can be distinguished by different colors.

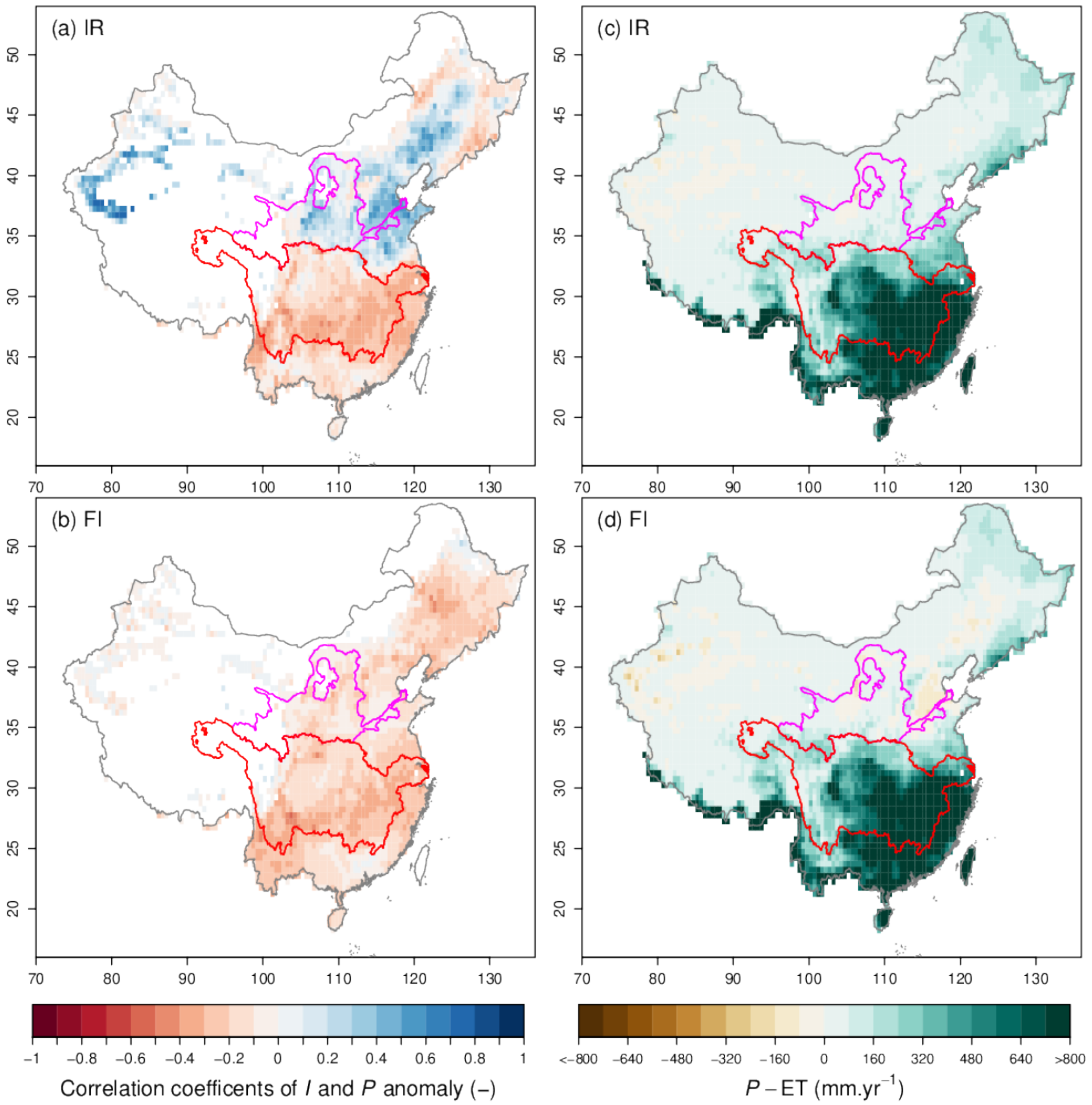

Figure S8. (a)-(b): Correlation coefficients between monthly irrigation anomaly and precipitation anomaly. (c)-(d): Differences between mean annual P and simulate ET.

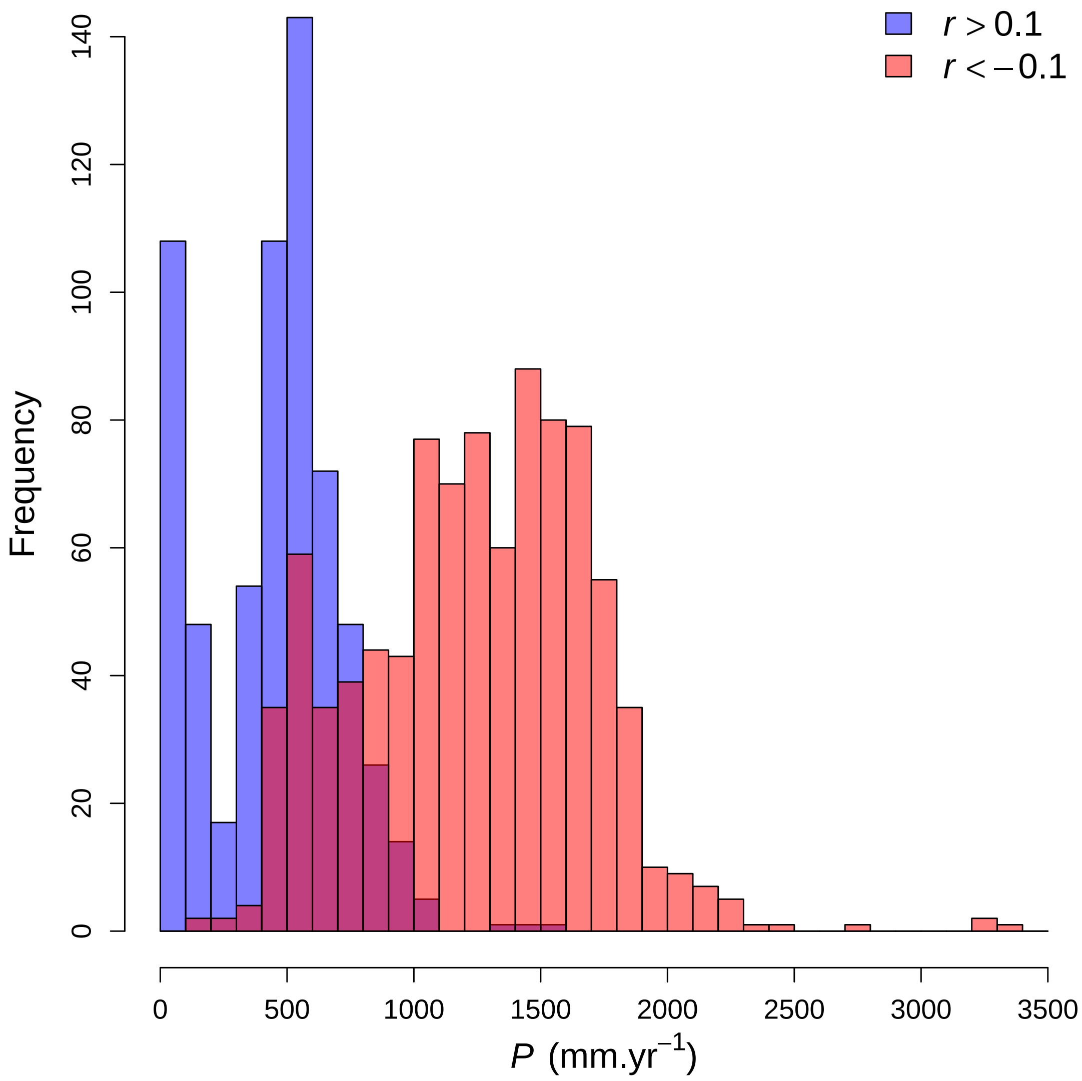

Figure S9. Histograms of correlation coefficients (r) between I and P anormaly based on the IR simulation (Fig. S8(a)). Blue accounts for positive r, while red accounts for negative r.

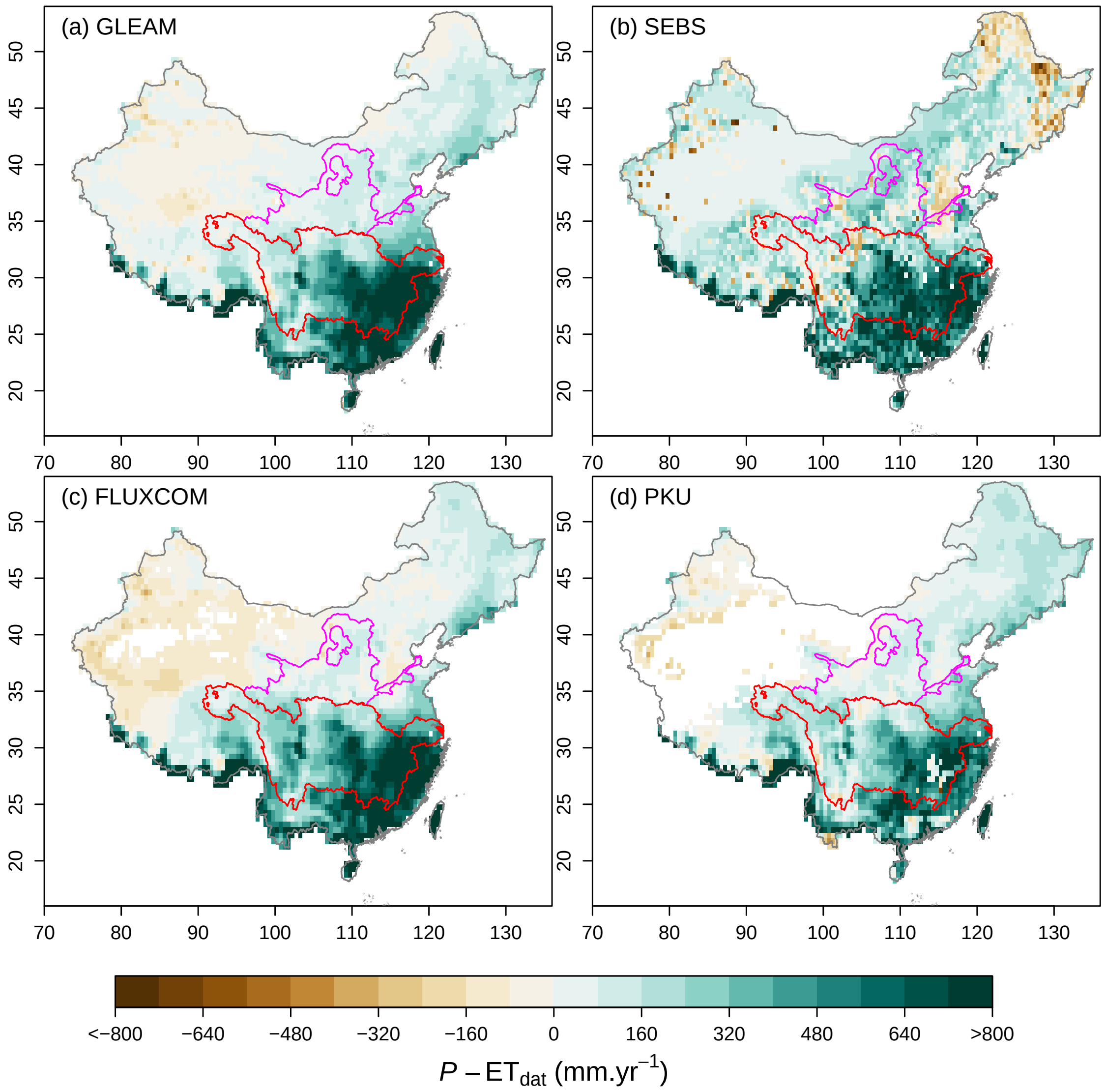

Figure S10. Differences between GSWP3 mean annual P and mean annual ET from multiple products.

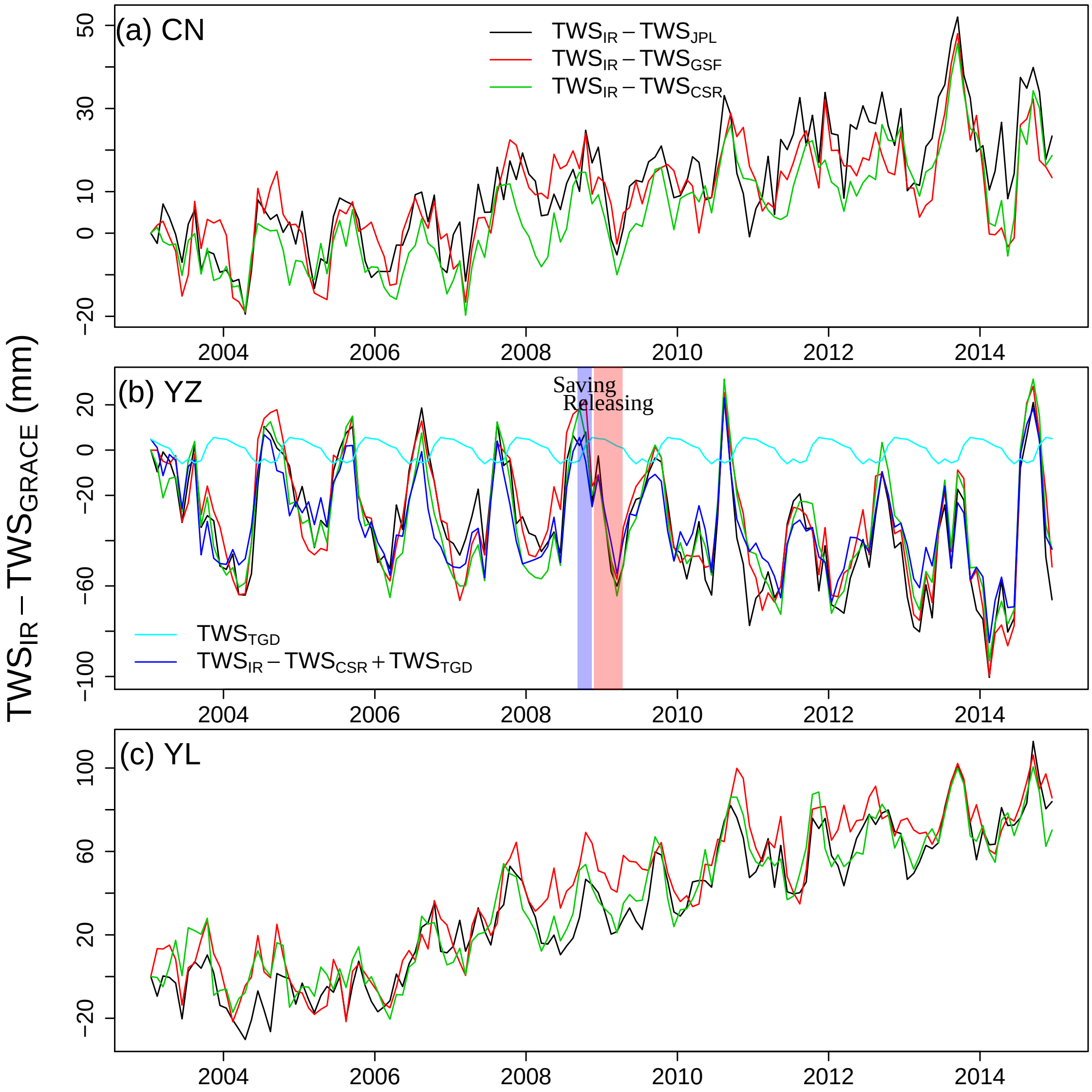

Figure S11. Difference between simulated and GRACE TWS in different regions. We assume that the values of simulated and GRACE TWS are the same at the first time step of the overlay period. In (b), cyan line indicates pesudo contributions of the Three Gorges Dam (TGD) to TWS (TWS_{dam}, Sect. 2.2.3). Blue line indicates the difference between TWS_{IR} and TWS_{CSR} corrected by TWS_{dam}. Blue and red areas indicate the periods of water recharging and releasing of the TGD, respectively.

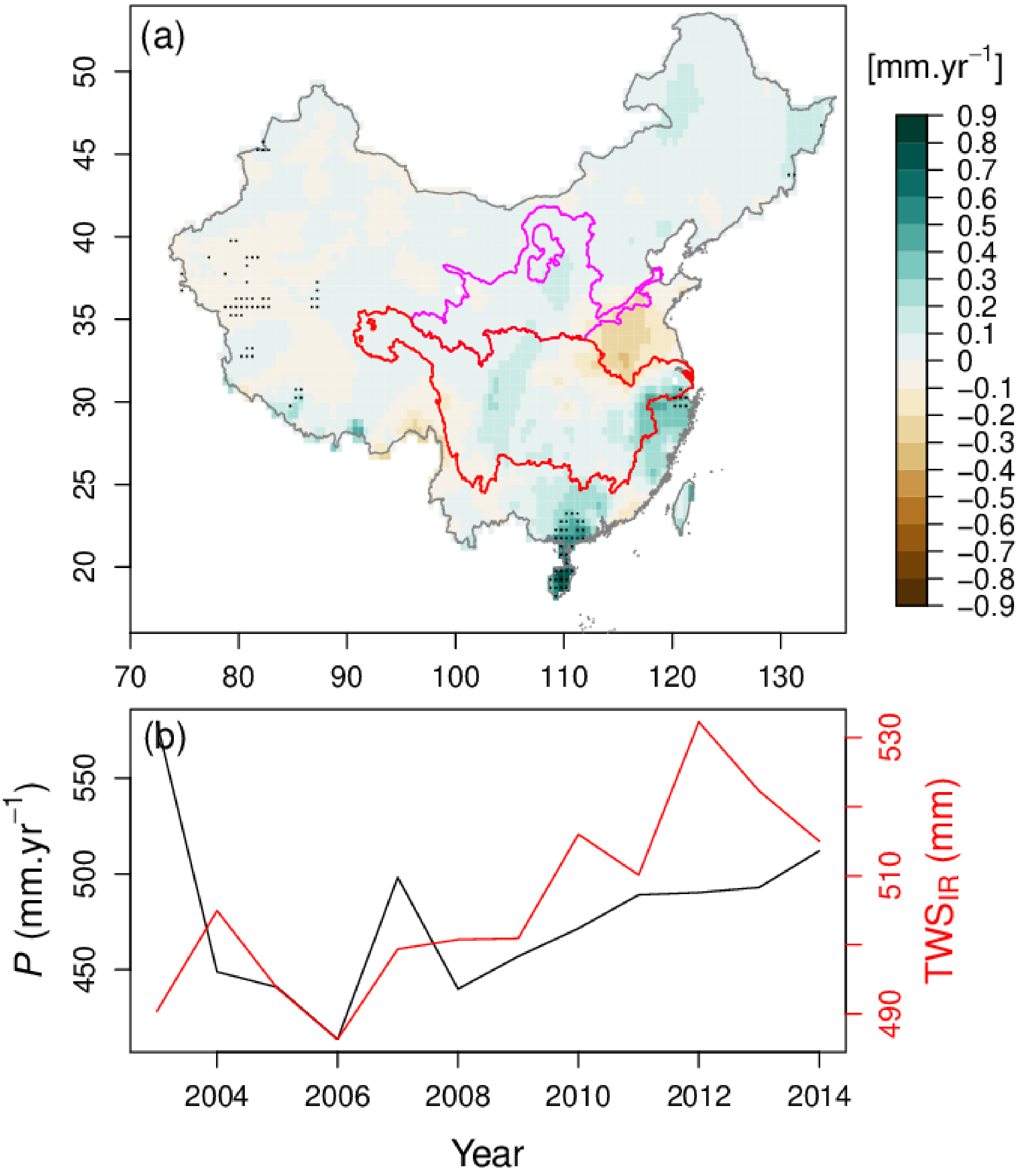

Figure S12. (a) Trend of P from 2003-2014. Black points indicate significant trend ($p < 0.05$) according to Mann-Kendall test. (b) Time series of annual P and TWS_{IR} in YLRB.

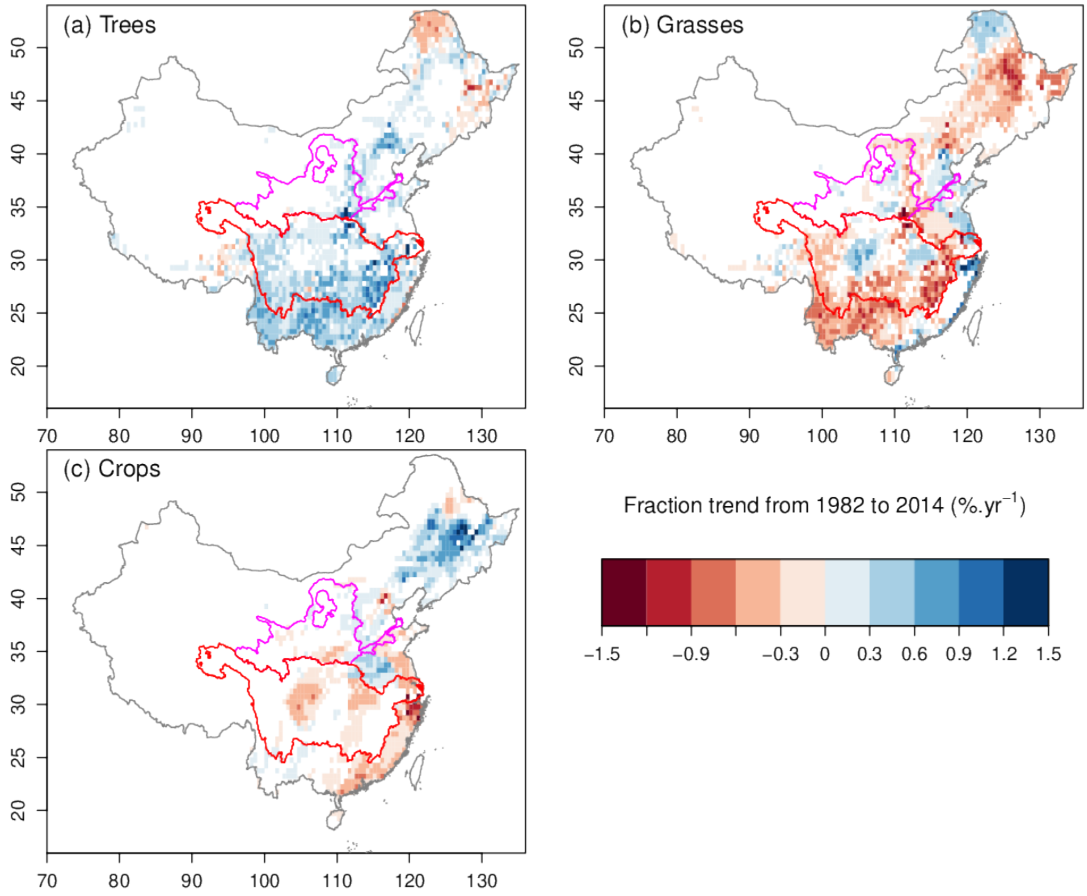

Figure S13. Trends of fractions of trees, grasses, and crops from 1982 to 2014 based on the 15-PFT map. Grid cell is colored if its p-value < 0.05 according to Mann-Kendall test.

Table S1. Information of the four ET datasets used in this study.

Dataset	Resolution	Duration	Algorithm	SM observations
GLEAM	0.25°, daily	1980–2017	Priestley-Taylor	ESA CCI
FLUXCOM	0.5°, monthly	1982–2011	FLUXNET & LPJmL	NA
SEBS	0.1°, daily	2000–2017	Surface energy balances	NA
PKU	0.5°, monthly	2000–2017	Water balances	NA

Table S2. Plant Functional Types of the land cover map used in this study and related soil tiling scheme. Gray indicates that the specific PFT belongs to corresponding soil tile.

PFT Index	Plant Functional Types	Soil tile index						
		1	2	3	4	5	6	7
1	Bare ground	■						
2	Tropical broad-leaved evergreen		■					
3	Tropical broad-leaved raingreen		■					
4	Temperate needleleaf evergreen		■					
5	Temperate broad-leaved evergreen		■					
6	Temperate broad-leaved summergreen		■					
7	Boreal needleleaf evergreen		■					
8	Boreal broad-leaved summergreen		■					
9	Boreal needleleaf summergreen		■					
10	C3 grass			■				
11	C4 grass			■				
12	Wheat				■			
13	Maize					■		
14	Rice						■	
15	Other crops							■

Table S3. Statistical provincial water withdrawal according the the Chinese Year Book from 2003 to 2014 (`http://www.stats.gov.cn/tjsj/ndsj/`). Provinces listed below are mainly located over the Yellow River Basin. The unit is $10^8\,\mathrm{m}^3.\mathrm{yr}^{-1}$.

Year	Ground water withdrawal					Surface water withdrawal				
	Qinghai	Gansu	Ningxia	Shaanxi	Shanxi	Qinghai	Gansu	Ningxia	Shaanxi	Shanxi
2003	4.45	28.21	5.95	36.17	33.82	22.57	93.98	75.57	20.84	43.53
2004	5.1	28.1	6.5	35.8	32.9	23.9	93.1	57.6	19.9	40.6
2005	7.0	28.2	5.4	35.0	32.9	23.2	93.2	68.7	20.4	41.9
2006	7.0	29.5	5.4	34.8	33.5	23.7	92.3	72.7	20.5	44.4
2007	7.5	28.5	5.0	37.7	34.8	24.7	92.7	72.6	21.5	48.6
2008	7.7	28.8	5.1	36.2	33.4	23.5	91.9	65.9	22.5	47.3
2009	10.1	27.7	5.1	35.1	34.4	24.1	92.5	69.0	21.8	50.2
2010	4.7	24.0	5.2	32.9	33.1	23.9	94.7	67.0	23.3	50.9
2011	5.0	24.2	5.4	34.5	33.3	25.6	96.1	67.0	29.3	49.5
2012	5.0	24.2	5.4	34.5	33.3	25.6	96.1	67.0	29.3	49.5
2013	3.5	25.7	5.5	38.8	33.4	23.8	95.9	63.8	31.8	54.0
2014	3.8	29.4	5.6	36.1	33.5	24.3	90.9	66.4	33.2	54.6
Sum	70.9	326.6	65.4	427.6	402.4	288.8	1123.4	813.2	294.5	575.0
